# Epstein-Barr virus-infected tonsillar marginal zone B cells *in vivo* as a precursor for immunosuppression-related B-cell lymphoma

**DOI:** 10.1128/jvi.01051-24

**Published:** 2025-07-08

**Authors:** Charles Torgbor, David A. Thorley-Lawson, Ann M. Moormann

**Affiliations:** 1 Tufts University School of Medicine 12261, Boston, Massachusetts, USA; 2 Department of Medicine, University of Massachusetts Medical School 164186, Worcester, Massachusetts, USA; University of Toronto, Toronto, Ontario, Canada

**Keywords:** Epstein-Barr virus, B cells, B-cell lymphoma

## Abstract

The germinal center model (GCM) of Epstein-Barr virus (EBV) persistence in humans proposes that EBV infects and drives naïve B cells through the GC to establish a quiescent lifelong persistent infection in memory B cells. EBV causes several cancers, and the normal *in vivo* counterparts of major EBV-associated cancers have been identified in the GCM. Yet, the normal *in vivo* counterparts of immunosuppression-related/immunoblastic B-cell lymphoma (IL), which occurs in immunosuppressed transplant and HIV patients and resembles lymphoblastoid cell lines (LCLs), remain elusive. We performed a comparative analysis of human tonsillar B cells to identify the closest normal *in vivo* counterparts of ILs/LCLs. Here we show that EBV-infected tonsillar marginal zone (MZ) B cells, which are GC-independent, are the closest *in vivo* correlates of ILs/LCLs. Like LCLs, EBV-infected MZ B cells express the EBV growth program, which includes high expression of the oncogenic LMP1 and the DNA-mutating enzyme activation-induced cytidine deaminase (AID), in the absence of the transcription factor BCL6. Furthermore, these EBV-infected MZ B cells are proliferating extensively and are Ki67^+^. AID expression, rapid proliferation, and LMP1 expression put these cells at high risk for tumor development. Of therapeutic importance, the high expression of LMP1 could prove to be the Achilles’ heel of IL/LCL-like EBV-infected MZ B cells to be targeted. Consequently, we now propose that there are two pathways of EBV persistence *in vivo*: (i) the established GC model and (ii) the GC-independent MZ model, and EBV-infected MZ B cells are plausibly the *in vivo* precursors of IL.

**IMPORTANCE:**

Epstein-Barr virus (EBV) causes several cancers, and the normal counterparts of major EBV-associated cancers have been identified in the germinal center model (GCM) of EBV persistence in humans. We searched for the normal *in vivo* counterparts of immunoblastic B-cell lymphoma (IL) that occurs in immunosuppressed individuals. IL resembles *in vitro* EBV-infected B cells driven by EBV to become indefinitely proliferating lymphoblastoid cell lines (LCLs). Here, we demonstrate that EBV-infected tonsillar marginal zone (MZ) B cells, which are GC-independent, are the *in vivo* correlates of LCLs/ILs and the plausible precursors of IL in humans. EBV-infected MZ B cells express the EBV growth program with high expression of the oncogenic LMP1 and the DNA-mutating enzyme AID and are proliferating extensively. AID expression, rapid proliferation, and LMP1 expression put these cells at high risk for cancer development. Clinical interventions that target LMP1-expressing MZ B cells should dramatically decrease the incidence of IL.

## INTRODUCTION

Immunosuppression in transplant or HIV-infected patients can increase the risk of lymphoma. Although these are a heterogeneous group of tumors, many are immunoblastic lymphomas (IL) arising from activated B cells and containing Epstein-Barr virus (EBV), which is a major etiological factor (1–7). Indeed, approximately 200,000 new cases of some major EBV-associated cancers occur worldwide yearly (8).

EBV is a γ-herpes virus that infects, establishes, and maintains a largely asymptomatic (9) persistent infection in more than 90% of the world’s population. The germinal center model (GCM) of EBV persistence describes EBV biology in humans. According to the GCM, EBV uses normal B-cell biology to establish and maintain persistent infection in memory B cells, where the virus resides in a quiescent state for the lifetime of the host. *In vivo*, newly infected naïve B cells are driven by EBV to become activated B lymphoblasts (10). This is the growth program (Latency III) (11–13) and includes nine latent proteins (6 EBV nuclear antigens [EBNAs] and 3 latent membrane proteins [LMPs]) under the control of EBNA2, and several non-encoding RNAs, including EBV-encoded small RNAs (EBERs) as well as microRNAs (14). These activated B lymphoblasts are then allowed by EBV to differentiate into GC B cells which express the default program (latency II); this is typical of Hodgkin’s lymphoma/disease (HD) cells (15–18). Herein, LMP1 and LMP2 play crucial roles. LMP1 mimics constitutively active CD40, activating NF-κB, MAP kinase, and interferon regulatory factor pathway (19, 20), thereby promoting growth and survival. Thus, LMP1 is oncogenic (21–23). LMP2, on the other hand, mimics tonic B-cell receptor signaling, activating PI3K/AKT, thus providing survival signals (20, 24, 25).

EBV-infected GC B cells then differentiate into memory B cells. Resting memory cells express latency 0, where there is no protein-encoding gene expression, allowing a non-pathogenic persistence of the virus. When memory B cells occasionally divide, the EBNA1-only program (latency 1) is turned on, and this is typical in Burkitt’s lymphoma (BL) cells (12, 26). Thus, potential precursors for BL and HD have been identified in the GCM (27). However, the origins of ILs remain elusive (27).

For IL to arise, three events are required. First, the B cells must be infected with EBV and express the growth program (3, 5, 13, 27). Second, these infected cells must proliferate and accrue mutations that will further enhance the malignant phenotype (2, 28–32). Third, since infected cells are targets of the cytotoxic T cells (33, 34), the immune response must be suppressed. Steps 1 and 2 are mimicked by *in vitro* EBV infection of resting B cells (11, 28, 29). These EBV-infected cells grow rapidly (31, 35), ultimately giving rise to established lymphoblastoid cell lines (LCLs) that proliferate indefinitely.

These LCLs are characterized by the cellular phenotype of activated B cells, and they resemble IL (5, 30). Over time, LCLs acquire a progressively malignant phenotype (29). Thereby producing a fatal IL-like disease in immunocompromised mice (5, 36). The likely explanation is that LCLs accrue mutations over time as they proliferate (2, 28–32). A critical enzyme behind this occurrence is activation-induced cytidine deaminase (AID), which is expressed by LCLs (32, 37, 38). AID is a mutagenic enzyme, and it mediates the processes of somatic hypermutation (SHM) and class switch recombination (CSR) of immunoglobulin (Ig) genes in B cells, including GC B cells (39). AID also targets non-Ig genes (40, 41). If these mutations and resultant DNA breaks accumulate, they can result in lymphoma (42–46). If an LCL-type cell arises *in vivo*, it would be regulated by the immune response; hence, the final requirement for IL development is immunosuppression.

**Fig 1:**
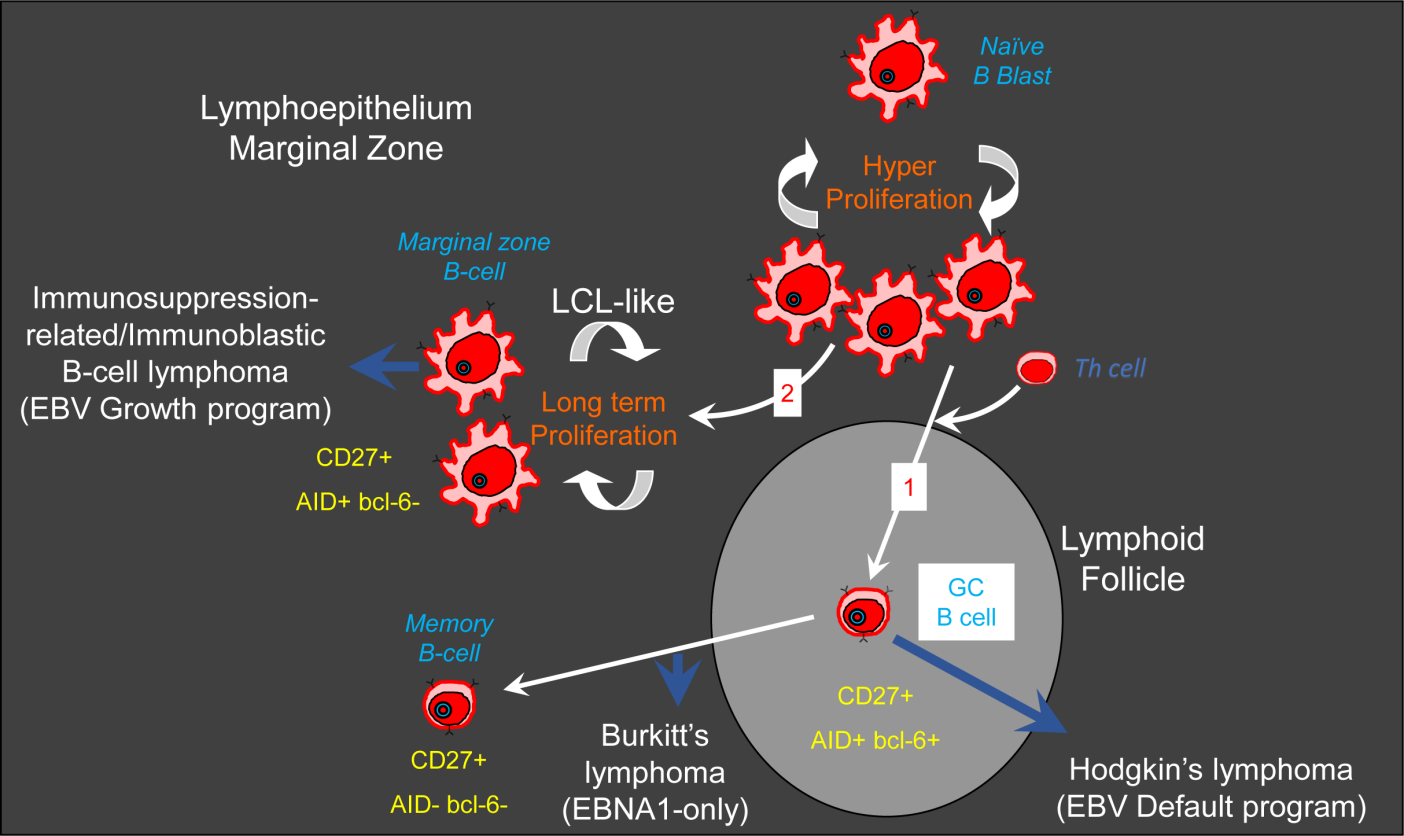
Two pathways/routes to EBV persistence and their associated lymphomas. Naive B cells become EBV-infected as they traverse the lymphoepithelium to the mantle zone, on their way from the high endothelial venules (HEV). EBV drives these cells to become activated B lymphoblasts. EBV-infected blasts migrate to the outer follicle due to their expression of the G-protein-coupled receptor EBI2. These cells express AID and experience SHM but are unable to enter the GC due to the lack of BCL6. Upon the receipt of requisite signals (including cytokines and/or T-cell help), they switch on BCL6, enter the follicle, and undergo the GC reaction. These EBV-infected cells are at risk of developing into Hodgkin’s disease/lymphoma. Latently infected GC B cells eventually exit as memory B cells as described in the GCM. Infected GC B cells that are unable to finally exit as resting memory B cells due to accrued mutations and genetic translocation (c-Myc/Ig) can give rise to Burkitt’s lymphoma (Route 1; GCM). Failure to receive the requisite signal to switch on BCL6 means that EBV-infected B lymphoblasts will continue to proliferate as LCL-like CD27^+^ AID^+^ BCL6^−^ marginal zone B cells. It is possible that MZ B cells can attain a resting state and that activating factors can determine the balance between proliferating LCL-like EBV-infected MZ B cells (predominant) and resting EBV-infected MZ B cells. In the immunosuppressed, LCL-like EBV-infected MZ B cells can give rise to immunosuppression-related/immunoblastic B-cell lymphoma (Route 2; Marginal zone model). It is also possible that MZ B cells, including EBV-infected MZ B cells, gain access to the GC. NB: Route 1 of the model is established, and all stages have been confirmed with experiments *in vivo* in humans. Adapted from reference (27) with permission of Springer Nature BV.

We hypothesize that the normal counterparts of LCLs or ILs exist in healthy humans (27) (Fig. 1). LCLs derived from *in vitro* infection of naïve B cells express (27) AID in the absence of other markers of GC B cells, such as the GC-required master transcription factor BCL6 (32, 38, 47–49) or the cell surface marker CD10 (50). Furthermore, these LCLs express the Ig of naïve B cells, IgD, yet also express the cell surface marker, CD27, similar to marginal zone (MZ) B cells (51–53). Such LCL-like EBV-infected B cells have not been described in humans (Table S1). Human MZ IgD^+^ CD27^+^ B cells reside in the spleen and lymphoid tissue and are GC-independent (54, 55). They are BCL6^−^ (56), undergo SHM (52, 53, 55, 57); therefore, they must express AID at some place and time, but this remains controversial (58, 59).

In this study, we demonstrate that EBV-infected B cells plausibly bearing the hallmarks of LCLs/ILs exist in human tonsils obtained from healthy EBV carriers. These cells are EBV-infected MZ B cells. Just like LCLs or ILs, EBV-infected MZ B cells express the growth program with high levels of the oncogenic LMP1 and AID in the absence of BCL6. They are also proliferating rapidly. The significance of this study is that we have identified a new reservoir of EBV-infected cells that could give rise to the lymphomas that characteristically develop in immunosuppressed individuals. In doing so, we have identified these potential tumor precursors as a plausible target for therapeutic intervention. Finally, we provide the first explanation for the biological role of the LCL phenotype and identify the normal *in vivo* correlate of these cells. We thus describe and identify a novel site of EBV persistence, independent of the GCM.

## RESULTS

### EBV-infected cells are present in the MZ B-cell subset and other major tonsillar B-cell subsets

We hypothesized that, based on phenotype, IgD^+^ CD27^+^ tonsillar MZ B cells are the closest normal *in vivo* correlates of the *in vitro* IgD^+^ CD27^+^ IL-like LCL (27). We have shown previously that EBV-infected IgD^+^ B cells are present in human tonsils (60, 61). Yet, we now know that these IgD^+^ tonsil B cells consist of a mixture of truly naïve [IgD^+^ CD27^-^ (CD10^−^)] and MZ [IgD^+^ CD27^+^ (CD10^−^)] B cells.

**Fig 2:**
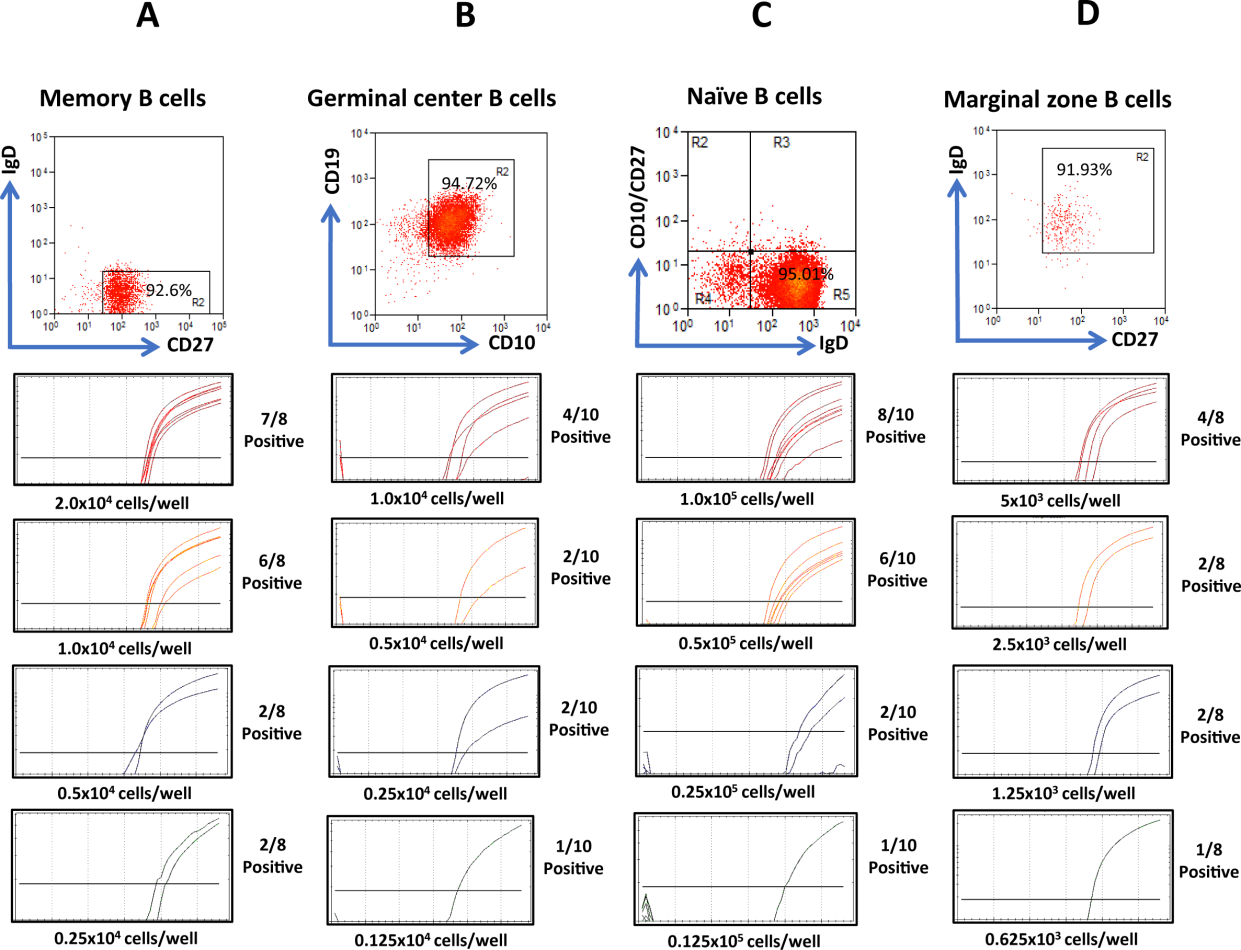
EBV is present at high levels in IgD^+^ CD27^+^ marginal zone B cells and other major B-cell subsets of the tonsil. (**A**) Memory B cells contain EBV. IgD^−^ CD27^+^ memory B cells were isolated by FACs and reanalyzed to check purity by FACs. Limiting dilution DNA PCR analysis was carried out on these cells. Here, multiple replicates of independently generated cell-limiting dilutions for a given tonsil were tested for the presence of EBV with a real-time DNA PCR assay that can detect a single infected cell. The absolute frequency of EBV-infected cells was then calculated. The real-time PCR analysis is shown. It must be noted that any given real-time PCR amplification plot comprises independent replicates of samples that have the same cell number. (**B**) Germinal center B cells contain EBV. IgD^−^ CD27^−^ or CD19^+^ CD10^+^ germinal center B cells were isolated by FACS. Purity analysis and real-time PCR were as described in (**A**). (**C**) Naïve B cells contain EBV. IgD^+^ CD27^−^ CD10^-^ naïve B cells were isolated by stepwise column fractionation. CD10-positive cells were first depleted, followed by the depletion of CD27-positive cells, and then B cells were negatively selected. B cells were stained with IgD to check purity. Alternatively, naïve B cells were isolated, and purity was assessed by FACs analysis (for different/alternative strategies see Fig. S1). The limiting dilution real-time PCR analysis was as described in (**A**). (**D**) Marginal zone B cells contain EBV. IgD^+^ CD27^+^ marginal zone B cells were purified by FACs. Purity analysis and limiting dilution real-time PCR were done as described in (**A**).

We therefore asked if MZ B cells and truly naïve B cells are EBV-infected. To test whether MZ B cells and naïve B cells are EBV-infected, we isolated naïve B cells with the aid of an antibody-magnetic bead and column fractionation system (Fig. 2; Fig. S1). Alternatively, naïve B cells (Fig. S1) or MZ B cells (Fig. 2) were isolated concurrently by a combination of column fractionation and staining for IgD and CD27. The tonsil memory [IgD^−^ CD27^+^ (CD10^−^)] and GC (CD10^+^ or IgD^−^ CD27^−^) B-cell subpopulations were also tested for comparison. The frequency of EBV-infected cells in each population was determined using the limiting dilution DNA PCR method for the W-repeat region of EBV (WPCR). Our assay can detect a single EBV-infected cell (Fig. S1). The Poisson statistics was then applied to calculate the absolute frequency of EBV-infected cells. The results from nine tonsils are summarized in Table 1. In all cases analyzed, there were appreciable numbers of EBV-infected cells in all the tonsil B-cell subpopulations assayed. Our B-cell populations were always ≥90% pure and often ≥95% pure (Fig. 2; Fig. S1). Naïve B cells exhibited the lowest level of EBV-infected cells, whereas the level of EBV-infected MZ B cells was high, between that of the memory B cells (highest) and GC B cells (Fig. 2; Table 1).

**TABLE 1:** Frequency of EBV-infected cells in the tonsillar B-cell compartments^*a*^

Tonsil	Number of virus-infected cells/10^7^ B cells^*b*^			
	Memory	Germinal center	Naive	Marginal zone
1	2,255	1,140	268	1,880
2	2,689	1,251	234	1,313
3	2,759	662	192	1,032
4	1,106	503	34	443
5	ND	≥3,000	233	≥4,938
6	ND	ND	≥300	≥3,601
7	ND	ND	387	1,398
8	ND	ND	69	693
9	1,862	ND	67	ND

^*a*^ Each tonsil represents a different donor, and all tonsil codes, for example, 1, 2, 3, etc., refer to the same unique donor throughout this paper.

^*b*^ The number/frequency of EBV-infected cells was determined by limiting dilution DNA PCR as in Fig. 2. ND signifies not done, that is, not all B-cell compartments were tested for all tonsils.

Based on these results, we conclude that tonsillar memory B cells, GC B cells, naïve B cells, and our predicted normal *in vivo* counterparts of ILs/LCLs, that is, tonsillar MZ B cells, all contain appreciable levels of EBV-infected cells.

### EBV-infected tonsillar MZ B cells express the growth program

To determine whether EBV-infected MZ B cells are the closest normal *in vivo* counterparts of IL, we asked whether, like IL-like LCLs, MZ B cells express the EBV growth program genes. With the aid of real-time reverse transcriptase (RT) PCR, we first validated EBV gene expression assays by testing cell lines that utilize distinct EBV programs (Fig. S2). These assays detect EBV gene transcripts in one infected cell (Fig. S2). The LCLs IB4 and SP-LCL-LS express the EBV growth program (EBNA2^+^ LMP1^+^ LMP2^+^ EBNA1^+^ EBER1^+^ and EBNA1Qp^−^ [EBNA1 from the EBV Qp promoter]^−^), and the BL cell line, Rael, expresses the EBNA1-only program (Fig. S2 and S3). Our EBV gene expression assays are therefore sensitive down to a single infected cell and can also differentiate between different latency programs.

**Fig 3:**
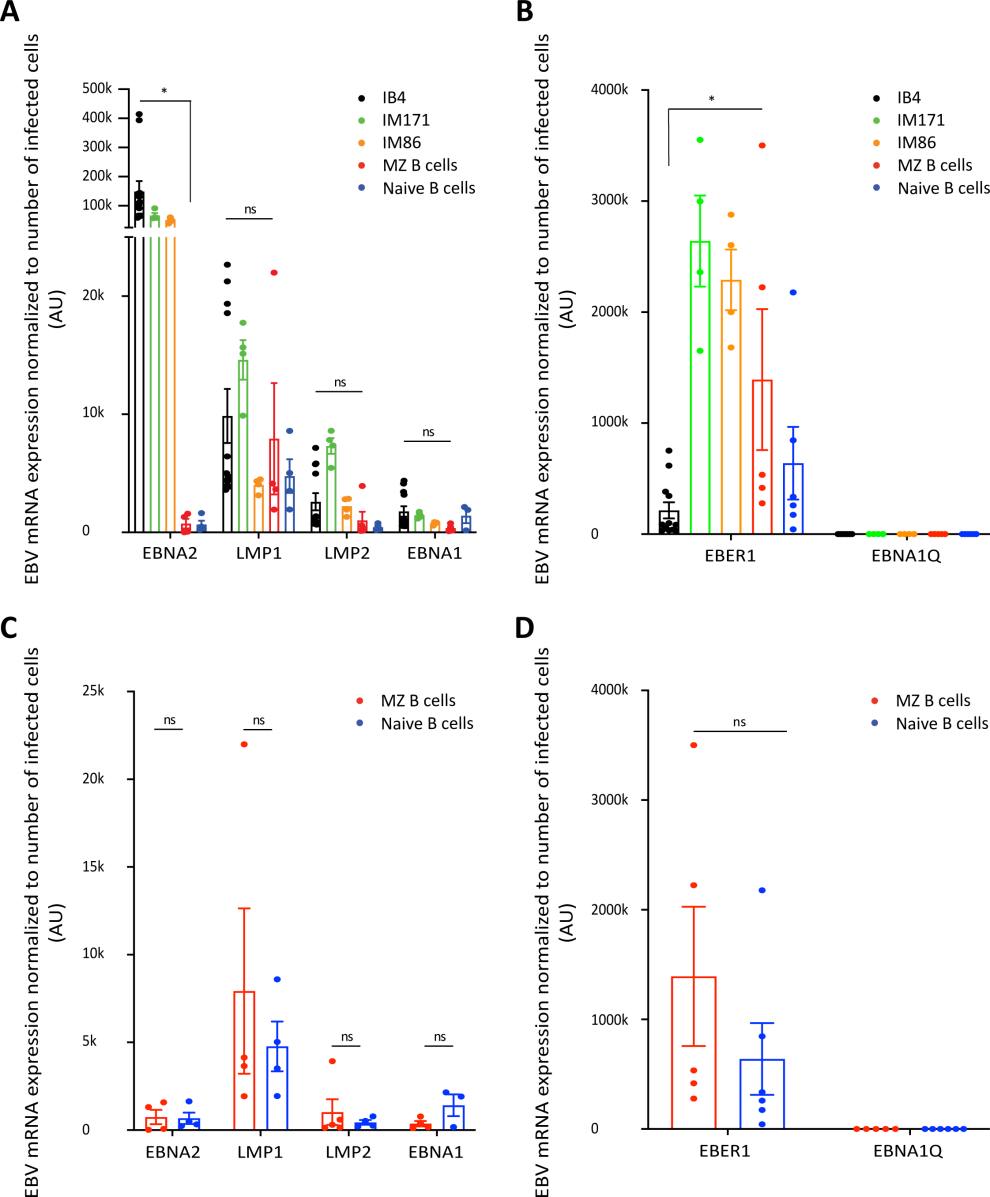
Tonsillar marginal zone B cells express the EBV growth program. (**A through D**) Tonsil marginal zone B cells (*n* = 5 tonsil donors) and tonsil naïve B cells (*n* = 6 tonsil donors) were isolated as indicated in Fig. 2 or Fig. S1. ~1 x 10^6^ tonsillar marginal zone B cells and ~2 x 10^6^ tonsillar naïve B cells per tonsil/tonsil donor were used per experiment. Multiple independent replicates of 200 cells from the standard lymphoblastoid cell line IB4 (*n* = 12) generated at independent/different times, or the spontaneously EBV-infected lymphoblastoid cell lines IM171 (*n* = 4) and IM86 (*n* = 4) were spiked into a standard number of EBV-negative tonsil cells or CB60-negative control cell lines before RNA extraction and synthesis of cDNA with Superscript IV First-Strand system (Invitrogen). EBV gene expression was analyzed by real-time RT PCR (Taqman) on each independent sample replicate in duplicates. The number of EBV-infected cells tested was derived from EBV frequencies. The frequencies were measured as in Fig. 2 and Table 1. (**C and D**) The EBV gene expression data for EBV-infected tonsil marginal zone B cells and EBV-infected tonsil naïve B cells in panels A and B have been highlighted in panels C and D, respectively. AU: arbitrary units. The level of EBV mRNA is expressed relative to cell number. Data are presented as mean ± S.E.M. *P < 0.05, ns represents not significant (two-tailed Student’s unpaired t test).

We then proceeded to perform real-time RT PCR for EBV gene expression on tonsillar naïve B cells and MZ B cells and asked whether EBV-infected MZ B cells express the growth program. Real-time RT PCR for EBV gene expression was also performed on the standard LCL IB4 and three spontaneously EBV-infected LCLs, IM171, IM86, and A.M(T2) for comparison. The standard LCL IB4 and 2 out of 3 spontaneously EBV-infected LCLs (within the limit of cell numbers assayed) express the EBV growth program (Fig. 3A and B; Fig. S2 to S4). Judging from 4 out of 5 tonsils analyzed, EBV-infected MZ B cells express the EBV growth program (Fig. 3A through D; Table 2; Fig. S2 to S4). It must be stated that, in the absence of the ability to perform absolute single-cell analysis on *in vivo* EBV-infected human B cells, there is a possibility that some EBV-infected MZ B cells may be able to exit the EBV growth program. EBV-infected naïve B cells also express the EBV growth program, albeit less frequently than MZ B cells (3 out of 6 tonsils), and the trend suggests a gradual turning on and perhaps shutting down of the EBV growth program. For example, the absence of EBNA2 and EBNA1Qp but the presence of LMP1, LMP2, and EBER1 could indicate that the growth program is being switched off concomitant with a tilt towards the default program of GC B cells. The presence of only EBNA2 and EBER1 could be indicative of the switching on of the EBV growth program (Fig. 3A through D; Table 3; Fig. S2 to S4). In MZ B cells (but not naïve B cells), since there is an association between the number of EBV-infected cells and the observance of EBNA2, the MZ B cell sample where only LMP2 and EBER1 are observed could be due to the cell numbers analyzed (Fig. 3A through D; Tables 2 and 3).

**TABLE 2:** EBV latent gene expression pattern in latently infected marginal zone (IgD^+^ CD27^+^) B cells from tonsils

Tonsil	No. of EBV^+^ cells tested^*a*^	EBV gene expression determined by real-time RT PCR (Taqman) analysis^*b*^					
		EBNA2	LMP1	LMP2	EBER1	EBNA1	EBNA1Qp
1	85	+	+	+	+	+	−
4	27	−	−	+	+	−	−
5	65	+	+	+	+	+	−
6	264	+	+	+	+	+	−
7	196	+	+	+	+	+	−

^*a*^ The number of EBV-infected cells tested is derived from frequencies that are expressed as the absolute number of EBV-infected cells per 10^7^ B cells in a given population. The frequencies were measured as in Fig. 2 and Table 1. Briefly, multiple independent replicates of independent limiting dilutions of marginal zone (IgD^+^ CD27^+^) B cells were made. This was followed by DNA PCR on each aliquot. The fraction negative for each dilution point was determined, and Poisson statistics was used to determine the EBV frequencies. From this frequency, the number of EBV-infected cells within any given population can be determined.

^*b*^ Real-time RT PCR analysis was performed as in Fig. 3. + signifies positive, that is, a particular gene transcript is present. − signifies negative, that is, a given gene transcript is absent.

**TABLE 3:** EBV latent gene expression pattern in latently infected naïve [IgD^+^ CD27^−^ (CD10^−^)] B cells from tonsils

Tonsil	No. of EBV^+^ cells tested^*a*^	EBV gene expression determined by real-time RT PCR (Taqman) analysis^*b*^					
		EBNA2	LMP1	LMP2	EBER1	EBNA1	EBNA1Qp
1	24	−	+	+	+	−	−
4	6	+	−	−	+	−	−
5	70	+	+	+	+	+	−
6	66	+	+	+	+	+	−
8	16	−	−	−	+	−	−
9	10	+	+	+	+	+	−

^*a*^ The number of EBV-infected cells tested is derived from frequencies that are expressed as the absolute number of EBV-infected cells per 10^7^ B cells in a given population. The frequencies were measured as in Fig. 2 and Table 1. Briefly, multiple independent replicates of independent limiting dilutions of naïve [IgD^+^ CD27^−^ (CD10^−^)] B cells were made. This was followed by DNA PCR on each aliquot. The fraction negative for each dilution point was determined, and Poisson statistics was used to determine the EBV frequencies. From this frequency, the number of EBV-infected cells within any given population can be determined.

^*b*^ Real-time RT PCR analysis was performed as in Fig. 3. + signifies positive, that is, a particular gene transcript is present. − signifies negative, that is, a given gene transcript is absent.

The EBV genes most highly expressed in EBV-infected MZ B cells were EBER1 and the oncogenic LMP1. The levels of LMP1 in the EBV-infected MZ B cells were comparable to that in the standard IB4 LCL (*P* = 0.692 two-tailed unpaired t test), whereas the levels of EBER1 were significantly higher in EBV-infected MZ B cells than in the IB4 LCL (*P* = 0.011 two-tailed unpaired t test). EBNA2 expression was significantly higher in the IB4 LCL (and 2 out of 3 spontaneous LCLs) compared to the MZ B cells (*P* = 0.034 two-tailed unpaired t test) (Fig. 3A through D; Fig. S3 and S4). The EBV-infected MZ B cells, naïve B cells, and all LCLs lacked EBNA1Qp (which is expressed by GC B cells) (Fig. 3A through D; Tables 2 and 3; Fig. S2 to S4). Curiously, we observed that the DNA tethering protein EBNA1, required to faithfully replicate the EBV genome (62, 63), was expressed at low levels. This corresponds with the observation that EBV microRNA limits the expression of EBNA1 (64) (Fig. 3A and C). Figure S3 shows representative PCR amplification plots of all the genes tested in EBV-infected MZ B cells, naïve B cells, and controls. It could be argued that the level of EBV gene expression in the cell lines we employed for our real-time RT PCR sensitivity testing assays might not reflect that of *in vivo* cells, and hence their use for sensitivity testing (Fig. S2) might not be apt. However, we could detect EBV gene transcripts in as few as 6 EBV-infected naïve B cells (Fig. 3A through D; Tables 2 and 3). In summary, just like IL-like LCLs, EBV-infected MZ B cells express the growth program.

### EBV-infected tonsillar MZ B cells have a history of extensive divisions

As opposed to the GCM, where EBV-infected naïve B cells proliferate briefly and then differentiate into GC B cells, we hypothesized that, like LCLs, EBV drives a subset of naïve B cells to become extensively proliferating LCL-like MZ B cells. We therefore proceeded to ascertain the history of proliferation of EBV-infected MZ B cells. We took advantage of the fact that as a newly EBV-infected B-cell population divides, the average viral genome copy number increases. We have previously reported that when EBV infects B cells *in vitro*, only one copy of a circular viral genome is retained (65, 66). This EBV genome divides over time as the infected cell divides to yield a heterogeneous population when grown in long-term culture. This population has a distinctive distribution that shows a predominance of cells with lower EBV genome copy number—put another way, most of the data points observed are towards the lower value range (18, 67). Thus, the EBV genome distribution profile provides us with information about the history of division of EBV-infected cells. This *in vitro* observation is a crude approximation of what occurs in EBV-infected cells *in vivo*, yet to date, this is the only method that gives information about the history of division of EBV-infected cells *in vivo*.

**Fig 4:**
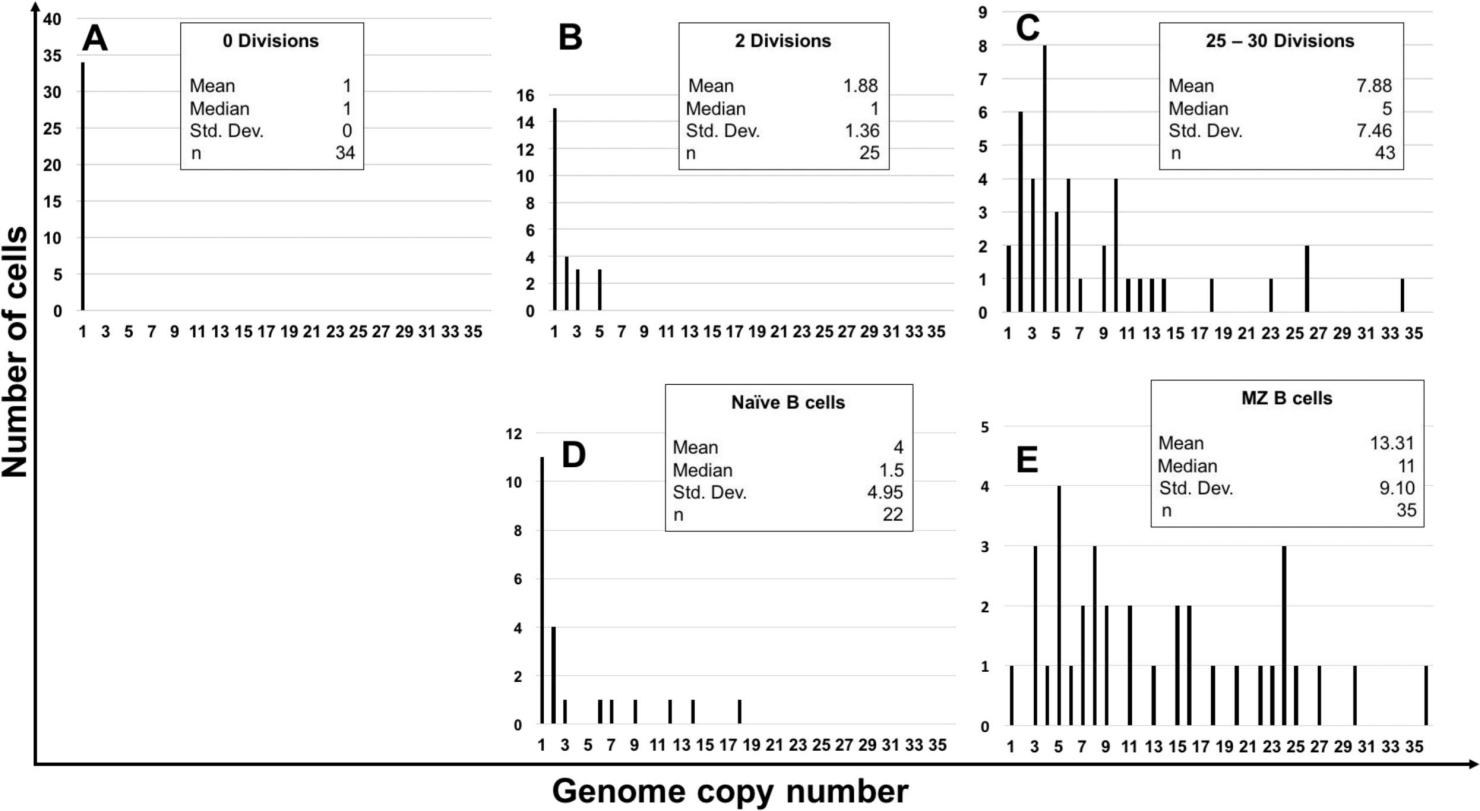
The distribution of EBV genomes in *in vitro* and *in vivo*-infected cells indicates that EBV-infected tonsillar marginal zone B cells have a history of extensive divisions. (**A**) *In vitro* newly infected peripheral blood B cells that had not yet divided based on carboxyfluorescein diacetate succinimidyl ester (CFSE) staining. (**B**) *In vitro* newly infected peripheral blood B cells that had undergone two divisions based on CFSE staining. (**C**) *In vitro* newly infected peripheral blood B cells that had been grown for 25 days (25–30 divisions). (**D**) The combined results of latently infected naïve B cells from five separate donor tonsils. (**E**) The combined results of latently infected marginal zone B cells from three separate donor tonsils.

Briefly, B-cell subpopulations were aliquoted such that there was one infected cell per well. WPCR was then performed on each well. The genome copy number was then estimated based on calibration curves from control cell lines (Namalwa Burkitt’s lymphoma cell line: 2 EBV copies per cell and IB4 lymphoblastoid cell line: 4 EBV copies per cell). The pattern of EBV genome copy distribution in newly *in vitro* infected peripheral B cells is demonstrated in Fig. 4A through C. These show the distribution of EBV genomes in newly infected peripheral B cells that have not divided (Fig. 4A), have undergone 2 (Fig. 4B), or 25 to 30 (Fig. 4C) divisions *in vitro*. We analyzed the EBV genome copy numbers in EBV-infected tonsillar naive B cells from five separate tonsils (Fig. 4D) and EBV-infected tonsillar MZ B cells from three separate tonsils (Fig. 4E). The distribution for EBV-infected MZ B cells contains a wide range of genome copy numbers (mean, 13.31; standard deviation, 9.10) and parallels cells that have undergone at least 25 divisions. The distribution of EBV-infected tonsillar naïve B cells indicates that one-half of the cells contain only one EBV genome, and the remaining contain mostly low genome copy numbers (mean, 4; standard deviation, 4.95) (Fig. 4). Thus, based on our analysis, EBV-infected tonsillar MZ B cells have a history of extensive division (≥25 divisions), whereas EBV-infected tonsillar naïve B cells have undergone few divisions.

### EBV-infected cells are enriched in the proliferating Ki67^+^ tonsillar MZ B-cell population

The limitation of our EBV genome copy approach is that it only describes the history of division of EBV-infected cells, but gives no idea of active proliferation. We reasoned that if latently infected tonsillar MZ B cells are the normal *in vivo* correlates of ILs and hence LCLs, then EBV-infected MZ B cells will be Ki67^+^. Ki67 is a proliferation-associated marker (68), and its presence indicates active proliferation.

**Fig 5:**
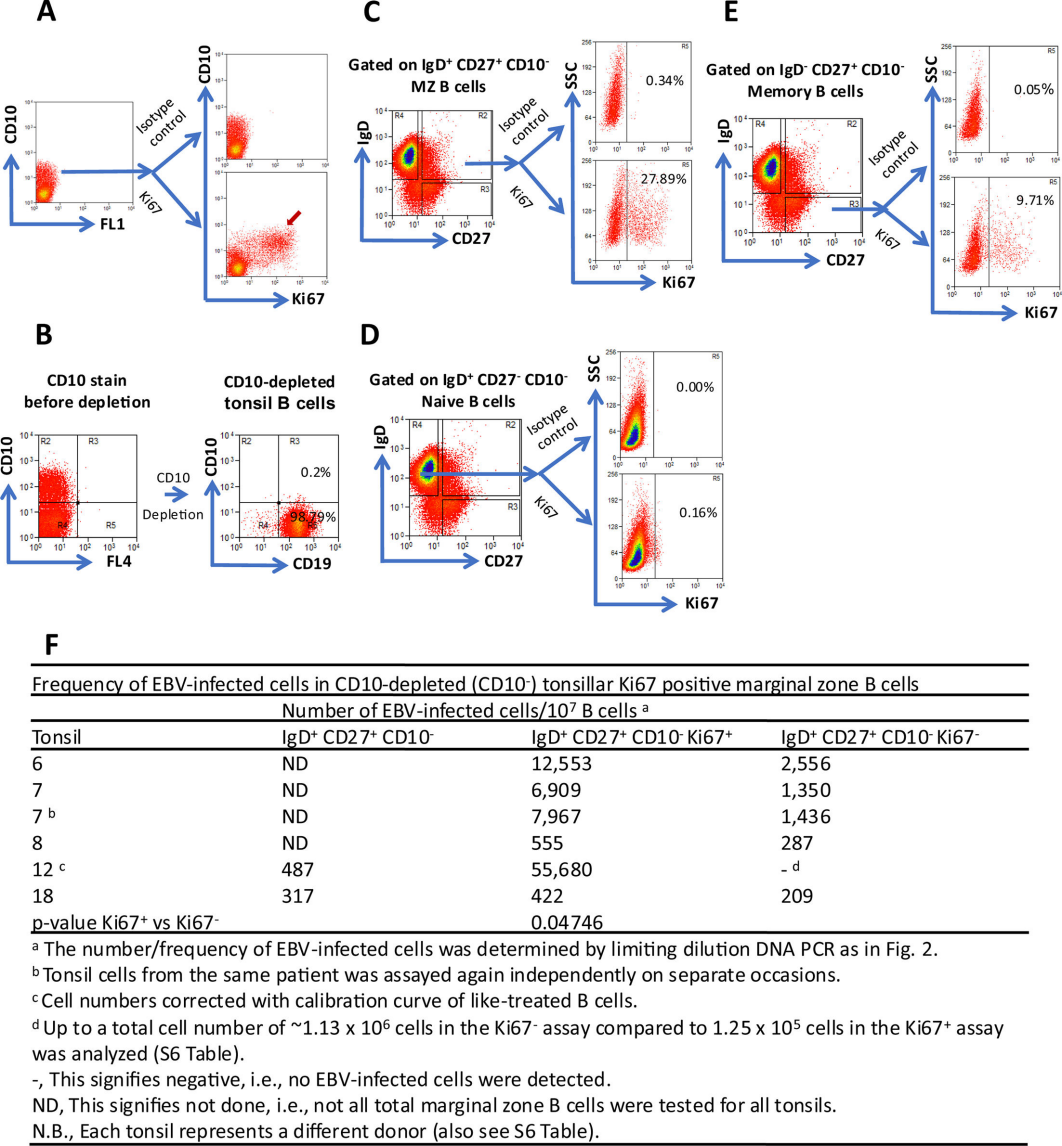
EBV-infected tonsillar marginal zone (IgD^+^ CD27^+^ CD10^−^) B cells are enriched in the proliferating population. (**A**) Flow cytometric analysis for the expression of the proliferation-associated marker Ki67 in tonsil GC (CD10^+^) B cells (positive control). Purified tonsil B cells were fixed, permeabilized, and stained for the intranuclear Ki67 (bottom) or isotype control (top). These cells were then washed and stained for the extracellular GC-specific marker CD10. The red arrow indicates Ki67^+^ GC B cells. (**B**) Efficient depletion of CD10^+^ cells, which express Ki67 from tonsil B cells by column fractionation. (**C**) CD10-depleted tonsil B cells, as in (**B**), were stained for the intranuclear marker Ki67 (bottom) or isotype control (top) and the extracellular markers IgD and CD27 as described in (**A**). Ki67^+^ and Ki67^−^ cells were then sorted from the IgD^+^ CD27^+^ CD10^-−^ marginal zone B-cell population. Total IgD^+^ CD27^+^ CD10^−^ marginal zone B cells were also sorted when possible. (**D**) CD10-depleted tonsil B cells stained as in (**C**) were gated on Ki67/isotype control expressing IgD^+^ CD27^−^ CD10^−^ naïve B cells (negative control). (**E**) CD10-depleted tonsil B cells stained as in (**C**) were gated on Ki67/isotype control expressing IgD^−^ CD27^+^ CD10^−^ memory B cells. (**F**) The frequency of EBV-infected cells in total marginal zone B cells, Ki67^+^, and Ki67^−^ marginal zone B cells was assessed based on limiting dilution DNA PCR. The Poisson statistics was applied, and then the absolute frequency of EBV-infected cells was calculated. Results of five different tonsils are shown. FL represents fluorescent channel. *P*-value was calculated using a one-tailed Mann-Whitney U test.

To test this hypothesis, we asked whether Ki67^+^ tonsillar MZ B cells are EBV-infected. CD10^+^ tonsillar GC B cells are highly proliferative and thus stain strongly for Ki67 as determined by intracellular Ki67 and extracellular CD10 flow cytometric analysis (Fig. 5A). With the aid of the antibody-magnetic bead and column fractionation system, we first depleted CD10^+^ cells from tonsil B cells with extremely high efficiency (Fig. 5B). This was followed by intracellular staining for Ki67 and extracellular staining for IgD and CD27. Our results show that in CD10^+^ cell-depleted (CD10-depleted) tonsils, MZ B cells stain strongly for Ki67, ~ 28% (Fig. 5C), indicating that they are highly proliferative, whereas naïve B cells hardly express Ki67 (Fig. 5D). Classical tonsillar memory B cells also express Ki67 but less so compared to MZ B cells (Fig. 5E). Ki67^+^ and Ki67^−^ MZ B-cell populations (Fig. 5C) were sorted, and DNA lysates of independent limiting dilution(s) were made. This was followed by WPCR to assess the level of EBV-infected cells in the resting (Ki67^−^) and proliferating (Ki67^+^) populations. The level of EBV-infected cells in total MZ B cells from such CD10-depleted cells was tested concomitantly in two (2) independent donor tonsils. The results are summarized in Fig. 5F. In five (5) out of five (5) independent CD10-depleted tonsils from separate donors, we found that Ki67^+^ MZ B cells are EBV-infected. Consistently, we found that EBV-infected cells were enriched in the Ki67^+^ MZ B-cell population. This result corresponds with a concomitant depletion of EBV-infected cells in the Ki67^-^ MZ B-cell population. In fact, in one tonsil sample, EBV^+^ MZ B cells were completely enriched in the Ki67^+^ population and entirely depleted from the Ki67^-^ population. It is noteworthy that, in one donor tonsil where we could perform the entire experiment on two separate occasions, there was a remarkable reproducibility of our technique (Fig. 5F). Due to the number of cells recovered from limited samples (e.g. ~5% to ~10% of tonsil B cells are MZ B cells), where necessary, we employed multiple independent replicates of single limiting dilution points. Nevertheless, our results are consistent and reproducible.

Our Ki67 results (Fig. 5) agree with our EBV genome copy results (Fig. 4). That is, EBV-infected MZ B cells have a history of multiple rounds of division (>25) and are proliferating, whereas EBV-infected naïve B cells have undergone few divisions. Thus, EBV-infected tonsillar MZ B cells have a history of extended division and are extensively proliferating, just like IL-like LCLs.

### A small fraction of tonsillar MZ B cells express AID, and EBV-infected tonsillar MZ B cells express AID

**Fig 6:**
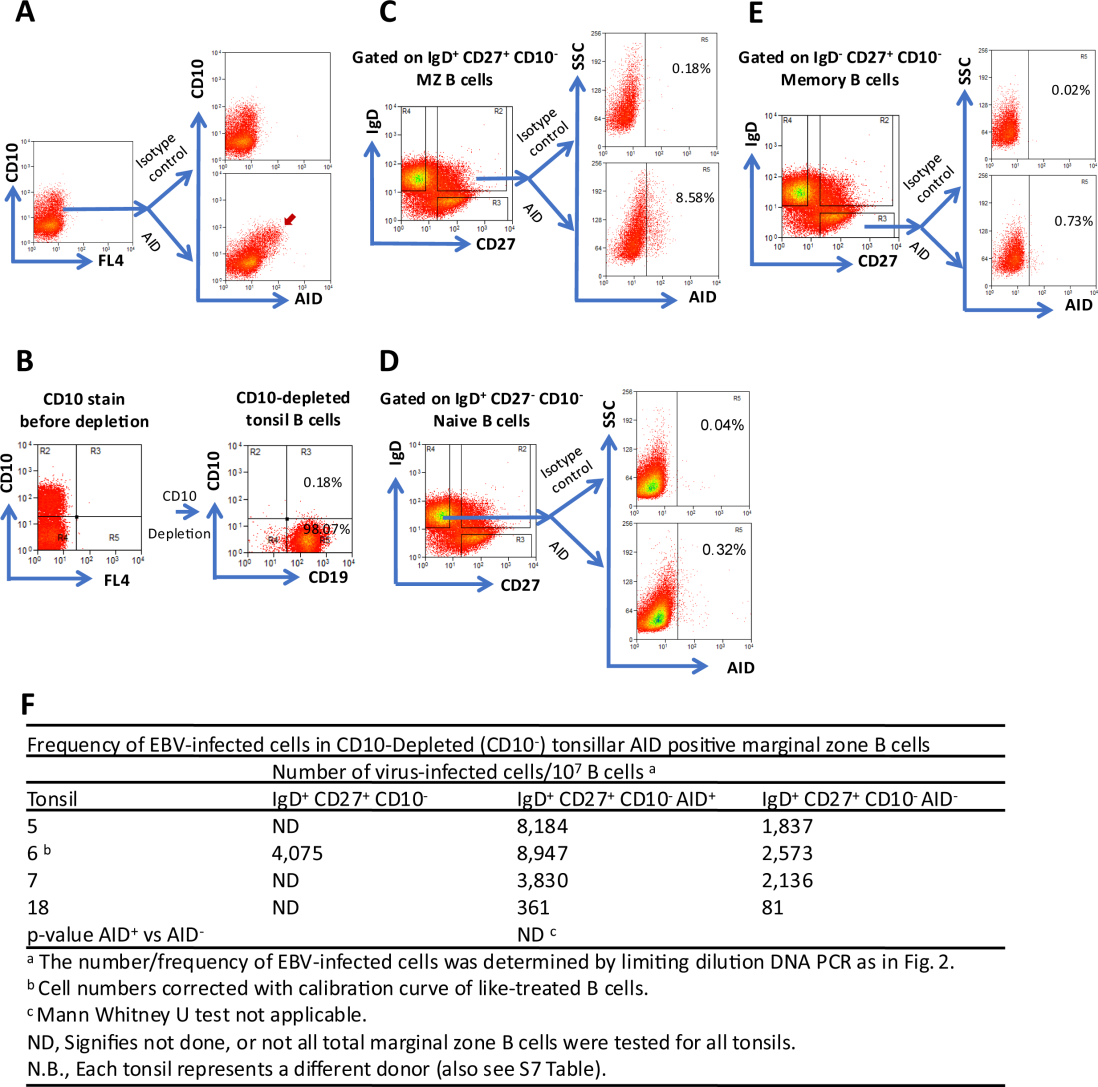
EBV-infected tonsillar marginal zone (IgD^+^ CD27^+^ CD10^−^) B cells express AID. (**A**) Flow cytometric analysis for the expression of the GC-associated mutagenic enzyme AID in tonsil GC (CD10^+^) B cells (positive control). Purified tonsil B cells were fixed, permeabilized, and stained for the intracellular AID (bottom) or isotype control (top). These cells were then washed and stained for the extracellular GC-specific marker CD10. The red arrow indicates AID^+^ GC B cells. (**B**) Efficient depletion of CD10^+^ cells, which express AID from tonsil B cells. (**C**) CD10-depleted tonsil B cells were stained for the intracellular AID (bottom) or isotype control (top) and the extracellular markers IgD and CD27 as described in (**A**). AID^+^ and AID^−^ cells were then sorted from the IgD^+^ CD27^+^ CD10^−^ marginal zone B-cell population. Total IgD^+^ CD27^+^ CD10^−^ marginal zone B cells were also sorted when possible. (**D**) CD10-depleted tonsil B cells stained, as in (**C**), were gated on AID/isotype control expressing IgD^+^ CD27^−^ CD10^−^ naïve B cells (negative control). (**E**) CD10-depleted tonsil B cells stained as in (**C**) were gated on AID/isotype control expressing IgD^-^ CD27^+^ CD10^−^ memory B cells. (**F**) The frequency of EBV-infected cells in total marginal zone B cells, AID^+^ and AID^−^ marginal zone B cells was assessed based on limiting dilution DNA PCR. The Poisson statistics was applied, and then the absolute frequency of EBV-infected cells was calculated. Results of four different tonsils are shown. FL represents fluorescent channel.

We hypothesized that, like IL-like LCLs, EBV drives a subset of newly infected naïve B cells to become extensively proliferating MZ B cells, which express AID. To test this hypothesis, we first asked whether tonsillar MZ B cells are AID^+^. CD10^+^ tonsillar GC B cells express AID as determined by intracellular AID and extracellular CD10 flow cytometric analysis (Fig. 6A). Therefore, with the aid of the antibody-magnetic bead and column fractionation system, we first depleted CD10^+^ cells from tonsil B cells (Fig. 6B). This was followed by intracellular staining for AID and extracellular staining for IgD and CD27. Our results show that a small fraction (~9%) of MZ B cells from CD10-depleted tonsils express AID (Fig. 6C). Neither tonsillar naïve B cells (Fig. 6D) nor classical memory B cells (Fig. 6E) express appreciable AID.

AID^+^ and AID^-^ MZ B-cell populations (Fig. 6C) were then sorted, and DNA lysates of independent limiting dilution(s) were made. This was followed by WPCR to assess the level of EBV-infected cells in the respective populations. The level of EBV-infected cells in total MZ B cells from such CD10-depleted tonsils was tested concomitantly in one donor tonsil. The results are summarized in Fig. 6F. In four (4) out of four (4) independent CD10-depleted tonsils from separate donors, we found that AID^+^ MZ B cells are EBV-infected. The frequency of EBV-infected MZ B cells from CD10-depleted tonsils was slightly enriched in the AID^+^ population (Fig. 6F), albeit not as pronounced as the Ki67 results (Fig. 5F). This result corresponds with a concomitant slight depletion of EBV-infected cells in the AID^−^ MZ B-cell population. Thus, EBV-infected tonsillar MZ B cells are AID^+^ just like IL-like LCLs.

### EBV-infected cells are enriched in the BCL6-negative tonsillar MZ B-cell population

**Fig 7:**
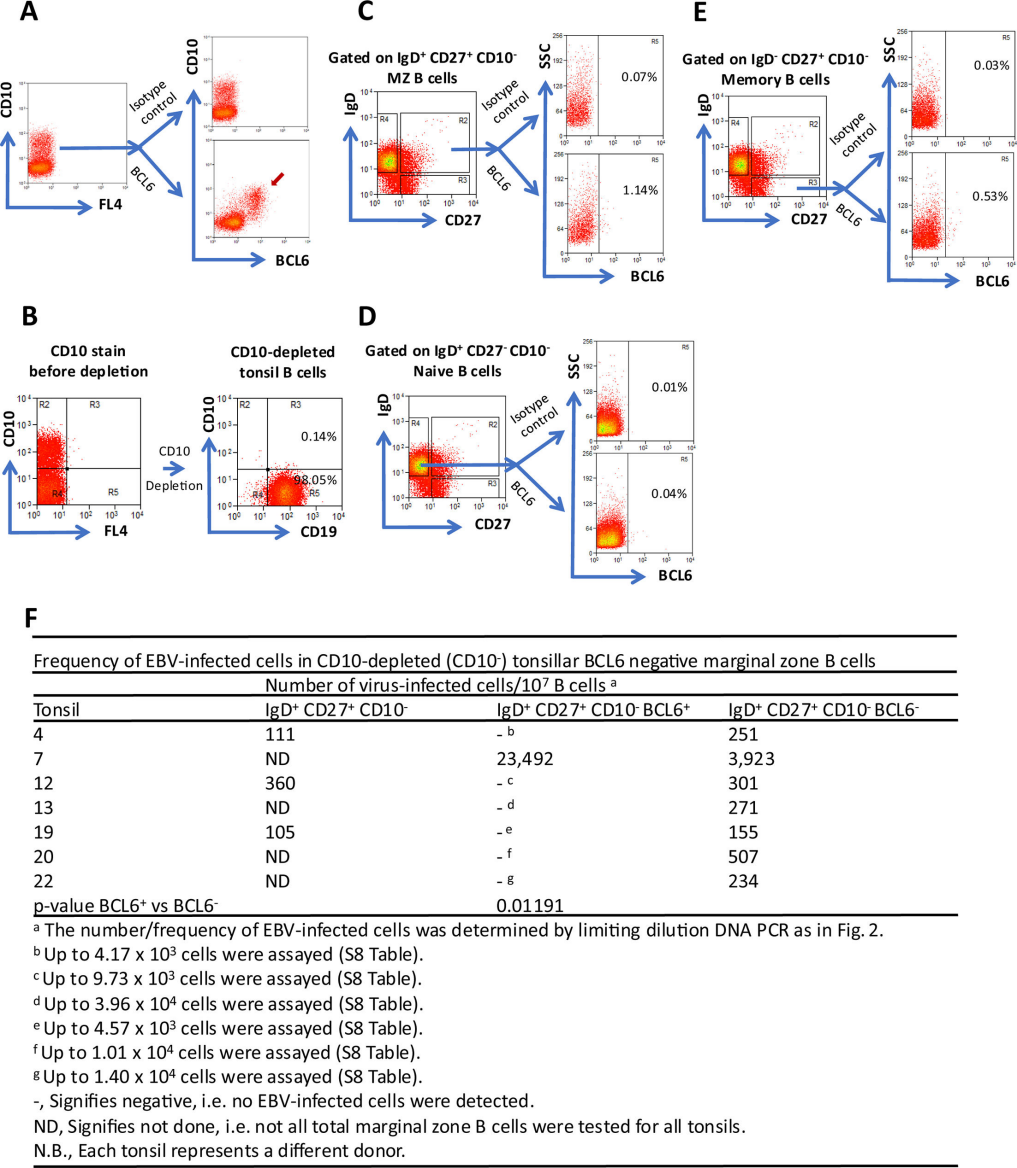
EBV-infected tonsillar marginal zone (IgD^+^ CD27^+^ CD10^−^) B cells are typically negative for BCL6. (**A**) Flow cytometric analysis for the expression of the GC-required transcription factor BCL6 in tonsil GC (CD10^+^) B cells (positive control). Purified tonsil B cells were fixed, permeabilized, and stained for the intranuclear BCL6 (bottom) or isotype control (top). These cells were then washed and stained for the extracellular GC-specific marker CD10. The red arrow indicates BCL6^+^ GC B cells. (**B**) Efficient depletion of CD10^+^ cells, which express BCL6, from tonsil B cells. (**C**) CD10-depleted tonsil B cells, as in (**B**), were stained for the intranuclear BCL6 (bottom) or isotype control (top) and the extracellular markers IgD and CD27 as described in (**A**). BCL6^+^ and BCL6^−^ cells were then sorted from the IgD^+^ CD27^+^ CD10^−^ marginal zone B-cell population. Total IgD^+^ CD27^+^ CD10^−^ marginal zone B cells were also sorted when possible. (**D**) CD10-depleted tonsil B cells stained as in (**C**) were gated on BCL6/isotype control expressing IgD^+^ CD27^−^ CD10^−^ naïve B cells (negative control). (**E**) CD10-depleted tonsil B cells stained as in (**C**) were gated on BCL6/isotype control expressing IgD^-^ CD27^+^ CD10^−^ memory B cells. (**F**) The frequency of EBV-infected cells in total marginal zone B cells, BCL6^+^, and BCL6^−^ marginal zone B cells was assessed based on limiting dilution DNA PCR. The Poisson statistics was applied, and then the absolute frequency of EBV-infected cells was calculated. Results of seven different donor tonsils are shown. FL represents fluorescent channel. *P*-value was calculated using a one-tailed Mann-Whitney U test.

If EBV-infected MZ B cells are the normal *in vivo* correlates of IL-like LCLs, they should be AID^+^, BCL6^−^ (32, 38), a phenotype currently unique to the LCL. CD10^+^ tonsillar GC B cells highly express BCL6 as determined by intracellular BCL6 and extracellular CD10 flow cytometric analysis (Fig. 7A). Thus, with the aid of the antibody-magnetic bead and column fractionation system, we first depleted CD10^+^ cells from tonsil B cells with extremely high efficiency (Fig. 7B). This was followed by intracellular staining for BCL6 and extracellular staining for IgD and CD27. Our results show that most, if not all, MZ B cells (~99%) are BCL6^−^ (Fig. 7C). Tonsillar naïve B cells (Fig. 7D) and classical memory B cells (Fig. 7E) are BCL6^−^. Next, we asked whether BCL6^−^ MZ B cells are EBV-infected. We sorted BCL6^−^ (predominant) and the rare, if any (or possible), BCL6^+^ MZ B populations from CD10-depleted tonsils. DNA lysates of independent limiting dilution(s) were made for onward WPCR to assess the level of EBV-infected cells. The level of EBV-infected cells in total MZ B cells from such CD10-depleted tonsils was tested concomitantly in three donor tonsils. The results are summarized in Fig. 7F. Our results indicate that in seven (7) out of seven (7) independent CD10-depleted tonsils from separate donors, BCL6^−^ MZ B cells are EBV^+^. What is remarkable is that, overall, the frequency of EBV-infected cells in the MZ B-cell population did not markedly change when counter-selected with BCL6. Furthermore, in six (6) out of seven (7) independent tonsils, we could not detect EBV^+^ cells in the rare, if any, BCL6^+^ MZ B cells retrievable.

Of note, we observed an exception to our finding in one donor tonsil. Herein, EBV^+^ MZ B cells were counter-selected with the few BCL6^+^ cells recovered (Fig. 7F). This is not entirely surprising because it has been reported that, *ex vivo*, upon CD40 ligand and Ig cross-linking, MZ B cells can be driven to induce BCL6 and “GC-like properties” (69). Indeed, MZ B cells have been proposed to interact with the GC at least in mice (70). Furthermore, studies in mice employing two-photon vital microscopy showed that the GC is a dynamic open structure, and non-GC cells occasionally pass through (71). These results suggest that MZ B cells (including EBV^+^) could gain access to the GC. This could have crucial implications for EBV persistence and EBV-driven disease.

First, it must be emphasized that these tonsils had been efficiently depleted of CD10^+^ cells (Fig. 7B), which otherwise express BCL6, the sine qua non of GCs, together with AID. Second, the percentage of AID^+^ and the rare BCL6^+^ MZ B cells do not coincide. Therefore, the simplest interpretation of our result is that these rare BCL6^+^ MZ B cells are a unique group of cells when and if ever present. Taken together, we conclude that just like IL-like LCLs, EBV-infected tonsillar MZ B cells are Ki67^+^, AID^+^, and BCL6^−^. Thus, our results consistently show that EBV^+^ tonsillar MZ B cells are plausibly the closest normal *in vivo* counterpart of the IL-like LCLs.

## DISCUSSION

The GCM has been instrumental in identifying the *in vivo* precursors of major EBV-associated cancers, including Hodgkin’s and Burkitt’s lymphoma (15–17, 26, 27, 72, 73). Yet, the plausible *in vivo* precursors of immunosuppression-related/immunoblastic B-cell lymphomas (ILs), which occur in the immunosuppressed (e.g., in transplant and HIV settings), remain elusive. These cancers resemble LCLs, have an activated B-cell phenotype expressing the EBV growth program, and can be recapitulated by LCLs in immunodeficient mice (4–6, 29, 30, 36).

Here, we report that in the tonsils of healthy EBV carriers, there exist EBV-infected cells that phenotypically resemble LCLs, and hence their biologically relevant correlates, ILs. We show that these are the EBV-infected tonsillar MZ B cells that are IgD^+^ CD27^+^ AID^+^ BCL6^−^ CD10^−^. Consistent with the LCL phenotype, we show that these EBV-infected MZ B cells express the EBV growth program, which will likely drive their proliferation. This agrees with our EBV genome copy distribution profile and Ki67 staining that indicate that EBV-infected MZ B cells have undergone extensive division and are actively proliferating.

In the absence of single-cell analysis on *in vivo* EBV-infected human B cells, it is possible that EBV-infected MZ B cells can eventually switch off all viral genes and attain a resting state (latency 0). This seems to be revealed by our Ki67 studies, where we found EBV-infected cells in both the MZ B cell Ki67^−^ (resting) as well as the Ki67^+^ proliferating populations, albeit EBV is remarkably enriched in the proliferating population. Thus, the following scenario is possible: EBV infects tonsillar naïve B cells and drives their activation and proliferation, and then one of two fates occurs. These EBV-infected naïve B cells either proceed through the GC reaction as described in the GCM or they differentiate into infected MZ B cells, a subset of which continue to proliferate (Fig. 1). This fits well with our Ki67 data and the fact that MZ B cells have an activated phenotype (74) and are rapidly activated *in vitro* (75). In fact, in one donor tonsil (1 out of 5 donor tonsils), all the EBV-infected MZ B cells were enriched in the proliferating population. What this could mean is that environmental cues could skew EBV-infected MZ B cells into the proliferating population (Fig. 1).

EBV-infected tonsillar naïve B cells also express the EBV growth program, but EBNA2 is less frequent in infected naïve B cells as compared to infected MZ B cells. A possible explanation is that EBNA2 expression in EBV-infected tonsillar naïve B cells is transient, and it is only required to establish a transient growth program/blast stage and then quickly allows the infected tonsillar B cell blast to differentiate. As adduced earlier, the pattern of EBV gene expression in EBV-infected naïve B cells suggests a gradual turning on of the EBV growth program, adding impetus to the existence of a pre-latent state (76), as well as a gradual turning off of the growth program. These observations are consistent with the low EBV genome copy profile, that is, limited division observed in the EBV-infected naïve B-cell population. Put another way, the EBV gene expression pattern and the genome copy profile indicate direct infection of naïve B cells, limited proliferation, and then differentiation. Combining our Ki67 and EBV genome copy profile results, the simplest interpretation is that naïve B cells are newly infected as opposed to the MZ B cells, which are derived/differentiated from EBV-infected naïve B cells. The MZ B cells continue to proliferate as observed by their expression of Ki67 and their EBV genome distribution profile. Thus, EBV-infected MZ B cells fit the LCL profile in being IgD^+^ CD27^+^ growth program^+^ with a history of many rounds of division as well as active proliferation, and hence are plausibly the normal *in vivo* counterparts of IL.

What then is the role of the potentially pathogenic EBV growth program in extensively proliferating EBV-infected MZ B cells? It is possible that, just as in established LCLs, EBV-infected MZ B cells maintain the ability to continue to express the growth program and proliferate since EBV latent proteins can function to specifically override cellular mechanisms that would under normal circumstances limit proliferation (76, 77). Alternatively, there could be activating factors that seem to favor the maintenance of the EBV growth program. The fact that MZ B cells display an activated phenotype (74) adds impetus to this alternate assertion. Moreover, the fact that cross-linking of Ig receptors unexpectedly induced EBNA2, and hence perhaps the “growth program” phenotype (78), means the activation of EBV-infected MZ B cells could possibly re-induce the growth program. Collectively, EBV and hence its specific growth program might just be exploiting the normal B cell biology of MZ B cells, inadvertently causing lymphoproliferative disease in the immunosuppressed.

EBV-infected MZ B cells also express the mutagenic enzyme AID. One caveat to our work is that we cannot tell whether the growth program expressing MZ B cells are at the same time Ki67^+^ and AID^+^. This is plausible since most of the EBV-infected MZ B cells are enriched in the Ki67^+^ population and also the AID^+^ population. That said, there is an oncogenic risk to proliferating EBV-infected lymphoblasts that express the mutagenic AID and also express the pro-survival BCL2 (Fig. S5) (79). They must be eliminated by EBV-specific cytotoxic T cells which, in fact, are found in tonsils (33) and respond to major EBV antigens (80, 81). It seems, however, that cytotoxic T cells might not be particularly effective in eliminating EBV-infected lymphoblasts *in vivo*, a mystery which remains to be fully resolved. Recent evidence indicates that EBV evades immune surveillance at least in part via the expression of EBV microRNAs (64, 82). These EBV microRNAs reduce immune surveillance by EBV-specific CD8^+^ T cells, suppress IL-12, and interfere with antigen processing (64, 82).

The failure of the immune system to eliminate EBV-infected lymphoblasts *in vivo* could also be explained by the action of LMP1, which is highly expressed in EBV-infected MZ B cells. LMP1 signaling through p38/PI3K pathways increases the expression of IL10 (83), which can inhibit EBV-specific cytotoxic T-cell activity (84). In addition, LMP1 induces the programmed cell death ligand 1 (PD-L1) (85). PD-L1 engages the PD-1 receptor on T cells, and this results in T-cell exhaustion. The high expression of the oncogenic LMP1 (21–23) in EBV-infected MZ B cells thus adds impetus to the oncogenic potential of these cells, especially in the immunosuppressed.

However, the seminal observation that LMP1 requires LMP2 to induce lymphoma in LMP1/LMP2 co-expressing transgenic mice could explain the selective development of lymphoproliferative disease in the immunosuppressed. There was little disease in immunocompetent mice; however, upon immunosuppression via the suppression of T cells and NK cells, there was a rapid and progressive fatal malignant phenotype (22). Critically, this implies that both the immune response and a precise modulation of EBV gene expression normally prevent the generation of EBV-associated lymphoproliferative diseases or IL. Notably, the fatal lymphoproliferative disease observed in LMP1/LMP2 co-expressing transgenic mice had a plasmablast phenotype (22). MZ B cells rapidly differentiate into plasmablasts upon encountering appropriate cues (70), and this could place differentiating EBV-infected MZ B cells at the epicenter of lymphoproliferative disease.

Our results plausibly bridge the connection between the GCM and the alternative direct-infection model proposed by Kuppers and Rajewsky. The direct-infection model proposes that EBV directly infects memory B cells (86). A key foundation of the direct-infection model is the observation that proliferating memory-like cells expressing EBNA2 exist in tonsils from infectious mononucleosis (IM) patients (86). These proliferating memory-like cells that express EBNA2 in the direct-infection model are likely the EBV-infected MZ B cells which are also memory-like (54, 75). The reason is that classical tonsillar memory B cells in the GCM do not express EBNA2 (15, 16) (Table S1). Moreover, our EBV genome distribution assay indicates that MZ B cells *in vivo* are plausibly not directly infected. Thus, the key tenet/foundation of the direct-infection model supports the proposed GC-independent route of EBV persistence shown herein (that is, proliferating memory-like EBV-infected MZ B cells expressing the EBV growth program), that originates from the direct infection of naïve B cells. Furthermore, unlike the GCM, the direct-infection model does not explain the four-distinct latent EBV gene expression programs at distinct stages of B-cell development (based on phenotypic and/or functional characteristics), or the origin of EBV-associated tumors (Table S1; Fig. 1). Intriguingly, recent elegant *in vitro/ex vivo* single-cell infection studies demonstrate that EBV can drive both GC and GC-independent-like phenotypes (87, 88), and that LCLs are a heterogeneous mixture of EBV-infected cells (89, 90) that include activated and plasmablast-like cells (89). These discoveries agree with our findings herein of the existence of the established GCM together with the alternate GC-independent MZ model of EBV persistence in humans.

Previous studies have remarkably attempted to analyze the origins of IL. First, LCLs transferred into immunodeficient mice give rise to IL (5, 29, 36). EBV growth program expressing cells that establish in humanized NOD-Scid γ_c_^-/-^ (NSG) mouse models give rise to IL-like disease upon T-cell depletion (91). Interestingly, the expression of LMP1 in B cells of transgenic mouse models mimics IL-like disease when T cells and NK cells are depleted (22, 23). The LMP1-transgenic mouse model mimics the LCL-transfer model of IL, seeing as LMP1 has been shown to induce NF-κB activation *in vitro* (19), and LCLs are crucially reliant on the LMP1-induced/NF-κB-induced pathway for survival (19, 92). Last, human IL but not BL is specifically characterized by constitutive NF-κB activation (4). These studies elegantly show why IL tumors arise but would not answer our question about the specific origin or plausible *in vivo* precursors of LCL-like IL versus BL or HD in humans.

It must be noted that the infection route in humanized EBV mouse models is intraperitoneal injection rather than the mouth (human route), and mice do not have tonsils where EBV persists. Hence, an amenable animal model for EBV persistence is lacking, and transgenic mouse models and *in vitro* studies are limited in their ability to specifically interrogate how IL arises. Consequently, our laboratory uniquely performs studies directly in the human host, which of necessity requires an approach wherein the hypothesis is tested iteratively. We have used this approach to analyze the origins of IL in humans. We confirm and extend attempts made in mouse models and *in vitro*, directly in human samples in the best way possible. That is, EBV-infected MZ B cells, which represent a GC-independent route of EBV persistence, are the closest *in vivo* correlates of LCLs/ILs and thus the plausible precursor of IL in immunosuppressed individuals.

Experiments that may provide further evidence that EBV-infected MZ B cells are the closest *in vivo* correlates of LCLs/ILs include the following very futuristic designs in *in vivo* human systems. (i) Comparing RNA-sequencing data of fresh IL samples with isolated EBV-infected MZ B cells versus uninfected MZ B cells versus LCLs. We hypothesize a constitutive NF-κB signaling signature (including upregulation of factors like c-FLIP that suppresses extrinsic apoptosis and necroptosis pathways) in EBV-infected MZ B cells, like what is observed in ILs/LCLs. (ii) Another feature, an aberrant somatic hypermutation signature seen in ILs/LCLs, may be observed in isolated EBV-infected MZ B cells. (iii) Finally, silencing the constitutive NF-κB signaling signature specifically in isolated EBV-infected MZ B cells would likely halt their growth, just like what is observed in LCLs/ILs. These designs will only be an extension, re-confirmation, or repetition of what we have already shown with the current data, employing systems yet to be developed for *in vivo* human work. To our knowledge, attempts have yet to be made to specifically isolate *in vivo* EBV-infected cells from chronic EBV carriers, and that is crucial to perform the futuristic experiments described above. Notably, these futuristic designs would also advance the approach described herein, which is limited in terms of unrestrictedly describing individual EBV-infected human B cells *in vivo* that express each of the EBV and host/human transcripts and proteins in question. That said, we may now begin to appreciate the regulation of EBV and host/human transcripts and proteins in EBV-infected B cells *in vivo* in humans, how this could engender disease(s), and how to prevent such disease(s) or design clinical interventions.

In conclusion, we have shown that EBV-infected MZ B cells in the tonsils of healthy human donors express the EBV growth program, have a history of extensive division, and are undergoing rapid proliferation while expressing the potent mutagen, AID. Proliferation and AID expression put these cells at high risk for the development of lymphoproliferative disease(s) in the immunosuppressed. In addition, conceivably, EBV-infected MZ B cells might be reliant on LMP1 just like LCLs (92), and this could prove to be the Achilles’ heel of these cells, LCLs, and ILs. We now propose that there are two pathways to EBV persistence *in vivo* in humans, that is, the established GCM and the alternate GC-independent MZ model of EBV persistence (Fig. 1), and EBV-infected MZ B cells are plausibly the normal *in vivo* precursors of the LCL-like IL.

## MATERIALS AND METHODS

### Cells, cell lines, and tissues

IB4 (gift of Dr. Elliott Kieff), an EBV-positive lymphoblastoid cell line, and Namalwa (American Type Culture Collection), an EBV-positive Burkitt’s lymphoma cell line were used as positive control for DNA PCR studies of the W-repeat region of the EBV genome. CB60 (gift of Dr. Miguel Stadecker), an EBV-negative mouse T-cell hybridoma cell line, and EBV-negative tonsillar mononuclear cells were used as negative control for all experiments. IB4 and SP-LCL-LS (gift of Dr. Alan Rickinson), a spontaneously EBV-infected lymphoblastoid cell line, were used as positive control for real-time RT PCR of EBNA2, LMP1, and LMP2. IB4 and Raji (gift of Dr. Jeffery Sample), an EBV-positive Burkitt’s lymphoma cell line, were used as positive control for real-time RT PCR of EBER1. IB4 was used as a positive control for real-time RT PCR of EBNA1. The EBV-positive Burkitt’s lymphoma cell lines Rael (gift of Dr. Samuel Speck) and Akata 2A8.1 (gift of Dr. Jeffery Sample) were used as positive control for real-time RT PCR of EBNA1Qp. The spontaneously EBV-infected lymphoblastoid cell lines IM171, IM86, and A.M(T2) (gift of Dr. Alan Rickinson) were tested for EBV mRNA expression.

The cell lines were cultured at 37°C with 5% CO_2_ in RPMI-complete medium. The RPMI-complete medium comprised RPMI 1640 supplemented with 10% fetal bovine serum (FBS), 2 mM glutamine, 2 mM sodium pyruvate, 100 IU of penicillin-streptomycin, and 10 µg/mL ciprofloxacin hydrochloride. For Akata 2A8.1 EBV-positive cell line, 300 µg/mL G418 selection antibiotic was added to RPMI-complete medium.

### Isolation of tonsil mononuclear cells

Tonsil mononuclear cells were isolated as described in detail previously (73). Briefly, tonsil mononuclear cells were isolated by mincing tonsil tissues in ice-cold 1× phosphate-buffered saline (PBS) + 0.5% bovine serum albumin (BSA) (PBSA). Ficoll-paque plus (GE Healthcare Biosciences, Philadelphia, USA) centrifugation was used to isolate tonsil mononuclear cells. The cells were re-suspended in FBS plus 10% dimethyl sulfoxide (DMSO) at 1 × 10^8^ cells/mL, aliquoted into cryotubes, stored on ice for about 5 minutes, and stored at −80°C overnight. The cells were then transferred to liquid nitrogen for long-term storage.

### Magnetic bead separations

To isolate tonsillar B cells, tonsillar mononuclear cells were resuspended in separation medium (StemCell Technologies, Vancouver, Canada). B cells were then purified using the StemSep B cell negative selection kit (StemCell Technologies, Vancouver, Canada) according to the manufacturer’s instructions. The purity of isolated B cells was tested by FACS analysis on the FACSCalibur. To isolate truly naïve B cells by antibody-bead column fractionation, tonsillar mononuclear cells were resuspended in RoboSep buffer (StemCell Technologies, Vancouver, Canada). CD10-positive and CD27-positive cells were sequentially depleted from tonsil B cells. This was achieved by sequentially staining tonsil cells with R-phycoerythrin (PE)-conjugated CD10 (CD10-PE) and CD27 (CD27-PE) antibodies (BD Biosciences, San Jose, USA). The PE selection kit with associated magnetic separation systems and the StemSep B cell negative selection kit with associated magnetic separation system (StemCell Technologies, Vancouver, Canada) were then used to deplete CD10^+^, CD27^+^, and non-B cells in stepwise fashion according to the manufacturer’s instructions. Work was done on ice. To deplete CD10^+^ cells from tonsil B cells, the same approach was used, except only the CD10-PE antibody (BD Biosciences, San Jose, CA, USA) was used. The isolated tonsil B-cell fractions were then resuspended in PBSA. The purity of CD10^−^ CD27^−^ tonsil naïve B cells or CD10-depleted tonsil B cells and the efficiency of the depletion process were then tested by FACS analysis on the FACSCalibur. To do this, aliquots collected at each step of the depletion process, along with aliquots of the negative fractions, were tested. The negative cell fractions were either left unstained or stained for CD19 (BD Biosciences, San Jose, CA, USA). For isolated naïve B cells, the negative cell fractions were also stained for IgD (Southern Biotechnology, Birmingham, AL, USA).

### Extracellular and intracellular staining

Tonsil B cells isolated as described above were resuspended at 1 × 10^6^ cells/100 µL PBSA for staining. For extracellular staining, the appropriate concentration of fluorochrome-conjugated antibody was added to cells and mixed gently but thoroughly. For concurrent separation of memory (IgD^−^ CD27^+^), germinal center (IgD^−^ CD27^−^), naïve (IgD^+^ CD27^−^), or marginal zone (IgD^+^ CD27^+^) B cells, tonsil B cells were stained with Alexa 647-conjugated anti-human IgD (Southern Biotechnology, Birmingham, AL, USA) and PE-conjugated anti-human CD27 (BD Biosciences, San Jose, CA, USA) antibodies. Alternatively, to isolate germinal center B cells, tonsil B cells isolated as above were stained with APC-conjugated anti-human CD19 and PE-conjugated anti-human CD10 antibodies (BD Biosciences, San Jose, CA, USA). For fixed cells, the PE-conjugated anti-human/hamster CD27 antibody from Miltenyi Biotec (Auburn, CA, USA) was used. The Alexa 488-conjugated anti-human IgD antibody (Southern Biotechnology, Birmingham, AL, USA) was used if extracellular staining was done after intracellular staining with an Alexa 647-conjugated anti-human antibody. The mixture was incubated for 15 minutes at room temperature in the dark. The cells were washed once with PBSA, vortexed gently, and spun down at 1,500 rpm at 4°C for 5 minutes. The cells were then resuspended in 300 µL PBSA and stored at 4°C until analysis.

To carry out intracellular staining, tonsil cells were fixed with BD Cytofix/Cytoperm fixation and permeabilization solution (BD Biosciences, San Jose, CA, USA), followed by incubation for 20 minutes at room temperature. The cells were then washed once with 1× BD wash/perm buffer (BD Biosciences, San Jose, CA, USA) and spun down at 1,500 rpm at 4°C for 5 minutes. A 0.5% saponin-based buffer was used to resuspend cells at 1 × 10^6^ cells/100 µL. An appropriate amount of normal human serum (Thermo Fisher Scientific, Rockford, USA) was added to block non-specific antibody binding. The cells were then incubated for 30 minutes at room temperature. Appropriate antibodies for intracellular antigens were added and incubated for 30 minutes at room temperature in the dark. The antibodies for intracellular antigens used were Alexa 647-conjugated anti-human activation-induced cytidine deaminase (AID) and isotype control; Alexa 647-conjugated anti-human BCL6 and isotype control; and FITC-conjugated anti-human Ki67 and isotype control (BD Biosciences, San Jose, CA, USA). The cells were washed once with wash/perm buffer and spun down at 1,500 rpm at 4°C for 5 minutes. The cells were then resuspended in 300 µL PBSA and stored at 4°C until analysis. Analysis was performed on a FACSCalibur, and cell sorting was performed on MoFLo, BD FACSAria, or Influx cell sorter at the Tufts University laser core. Sorted populations were always ≥90% pure and often ≥95% pure.

### Limiting dilution analysis and W-repeat EBV DNA PCR to determine the frequency of EBV-infected B cells

Limiting dilution analysis and W-repeat DNA PCR were performed as described in detail previously (73). Briefly, for limiting dilution analysis, specific tonsil B-cell subpopulations were isolated by the antibody-magnetic bead and column fractionation system and/or flow cytometry. Usually, about 8 –10 replicates each of specific dilutions, for example, 1 × 10^5^, 5 × 10^4^, 2.5 × 10^4^, 1.25 × 10^4^ cells (higher or lower dilutions were added as needed), were sorted or placed in a 96-well V bottom plate for subsequent EBV W-repeat DNA PCR. The cells were spun at 1,500 rpm at 4°C for 15 minutes, and supernatants were aspirated. Twenty microliters (20 µL) of digestion mix (10× PCR buffer, 100 µL; 4.5% Igepal (NP-40), 100 µL; 4.5% Tween-20, 100 µL; 20 mg/mL Proteinase K, 50 µL; and 650 µL of water; total volume of 1,000 µL) was added to each well and then incubated at 55°C overnight. Proteinase K was deactivated by heating at 95°C for 10 minutes and 10 µL of water was added to each well. DNA real-time PCR specific for the W-repeat sequence of the EBV genome was performed for each replicate as described previously (73). The frequency of EBV-infected cells in each B-cell population was calculated using Poisson statistics. See Tables S6 to S8 for the number of B cells employed for determining the frequency of EBV-infected cells in counterselection assays, and Table S9 for primers and probe sequences.

### Estimation of EBV genome copy number

The EBV genome copy assay was performed as described in detail previously (18). Briefly, the EBV genome copy number in tonsillar marginal zone B cells or naïve B cells isolated as described above, or newly infected B cells in culture, was determined. The frequency of EBV-infected cells in tonsillar marginal zone B cells and naïve B cells was determined by limiting dilution W-repeat DNA PCR as described above. Marginal zone B cells and naïve B cells were then sorted into 96-well plates at numbers predicted to give one infected cell per well. In all, three separate tonsils were analyzed for marginal zone B cells, and five separate tonsils were analyzed for naïve B cells. Newly infected cells were established with viral supernatant derived from the B95-8 cell line, and cells were labeled with CFSE (carboxyfluorescein diacetate succinimidyl ester) as described previously (18). The cells were harvested at various times after infection and analyzed for the number of times cells have divided by FACS based on CFSE labelling. Cells that had undergone 0, 2 divisions and cells that had been allowed to grow in culture for 25 days (25–30 divisions) were sorted singly into a 96-well plate. To determine the EBV genome copy number, DNA PCR for the W-repeat region of the EBV genome was performed as described above, and the signal obtained was compared to that obtained from a standard curve generated from cells with known EBV genome copy number. This curve was generated as follows: dilutions of IB4 (4 genomes/cell [93]) and Namalwa (2 genomes/cell [94]) cell lines were made by FACs. These contained five replicates each of 10,000 cells, 100 cells, 10 cells, 5 cells, 3 cells, 2 cells, and 1 cell. A plot of the Threshold cycle (C_T_) value from the quantitative DNA PCR against the log value of the respective cell number yields the standard curve.

### Purification of RNA and real-time RT PCR

RNA was isolated by TRIzol extraction (Invitrogen, Life Technologies, Grand Island, NY, USA). On average, ~1 × 10^6^ tonsillar marginal zone B cells and ~2 × 10^6^ tonsillar naïve B cells per tonsil/tonsil donor were used per experiment. DNA was eliminated by treating isolated RNA with TURBO DNase (Invitrogen, Life Technologies, Grand Island, NY, USA) before cDNA construction (where necessary). cDNA was constructed from RNA using the iScript cDNA synthesis kit (Bio-Rad, Hercules, CA, USA) as described previously (73). Alternatively, cDNA was made using the Superscript IV First-Strand system (Invitrogen, Life Technologies, Grand Island, NY, USA). A mixture of 5 µL random hexamers (50 ng/µL) and 1 µL deoxynucleotide triphosphates (dNTPs; 10 mM) was added to 6 µL of RNA and mixed. The components were briefly centrifuged at 1,500 rpm for 2 minutes and heated at 68°C for 8 minutes. This was followed by a 2 minute incubation at −20°C. The mix was rapidly centrifuged at 1,500 rpm for 2 minutes to remove condensation. Eight microliter (8 µL) of a mix containing 4 µL of 5× Superscript IV buffer, 2 µL of 100 mm dithiothreitol (DTT), 1 µL ribonuclease inhibitor, and 1 µL SuperScript IV reverse transcriptase (200 U/µL) (or 1 µL of water instead of reverse transcriptase for no reverse transcriptase control) was added. The reaction was centrifuged at 1,500 rpm for 2 minutes and incubated at 23°C for 10 minutes and 55°C for 50 minutes. The reaction was inactivated by incubating at 80°C for 10 minutes. The volume was brought up to 100 µL by adding 80 µL of RNA grade water. Real-time PCR was performed on synthesized cDNA for EBER1, EBNA2, LMP1, LMP2, EBNA1, and EBNA1Qp (see Table S9 for primers and probe sequences). Master mix for real-time PCR was prepared as follows: 12.5 µL SosoAdvanced Supermix (Bio-Rad, Hercules, CA, USA), 2.5 µL of primers (900 nM), and 2.5 µL of fluorogenic probe (250 nM). Twenty microliter (20 µL) of master mix was added to 5 µL of cDNA to make a final reaction volume of 25 µL. Real-time PCR was performed on the Bio-Rad iCycler or CFX96 Real-Time System, and the protocol was as follows: step 1, one cycle of 3 minutes at 95°C; step 2, 55 cycles of 15 seconds at 95°C and 1 minute at 60°C. The EBV gene expression assays detect mRNA from one EBV-infected cell (Fig. S2).

### Statistical analysis

The method used for determining statistical significance and *P* value is indicated in the figure legends where applicable. Statistical significance was determined by two-tailed unpaired Student’s t-test or one-tailed Mann-Whitney U test. Each tonsil code represents tonsils from an independent donor.

## Data Availability

All study data are included in the article and supplemental material.

## SUPPLEMENTAL MATERIAL

Supplemental material jvi.01051-24-s0001.pdf Supplemental text, Fig. S1 to S5, and Tables S1 to S9.

ASM does not own the copyrights to Supplemental Material that may be linked to, or accessed through, an article. The authors have granted ASM a non-exclusive, world-wide license to publish the Supplemental Material files. Please contact the corresponding author directly for reuse.
