# Supplementary material for: Epstein-Barr virus-infected tonsillar marginal zone B cells in vivo as a precursor for immunosuppression-related B-cell lymphoma
Source: J Virol. 2025 Jul 8;99(8):e01051-24. doi: 10.1128/jvi.01051-24 (PMC12363164; doi:10.1128/jvi.01051-24)
Supplement: Supplemental material — Supplemental text, Fig. S1 to S5, and Tables S1 to S9. [file jvi.01051-24-s0001.pdf]

Supplementary materials for

Epstein-Barr virus-infected tonsillar marginal zone B cells *in vivo* as a precursor for immunosuppression-related B-cell lymphoma

Charles Torgbor, [1*] David A. Thorley-Lawson, [1] and Ann M. Moormann [2*]

*Corresponding authors:

Charles Torgbor, charles.torgbor@gmail.com

Ann M. Moormann, ann.moormann@umassmed.edu

This file includes:

Supporting information text

Fig. S1 to S5

Table S1 to S9

SI references

David A. Thorley-Lawson and Ann M. Moormann contributed equally to this article.

David A. Thorley-Lawson passed away during the preparation of this manuscript.

Supporting Information Text

Limitations of Study

In summary, comparative analysis of human tonsillar B cells identified EBV-infected tonsillar marginal zone (MZ) B cells, which are germinal center (GC)-independent, as the closest normal *in vivo* counterparts of immunosuppression-related/immunoblastic B-cell lymphomas (ILs) and lymphoblastoid cell lines (LCLs). These EBV-infected MZ B cells plausibly express the EBV growth program—marked by high levels of the oncogenic LMP1, the DNA mutating enzyme AID, and are rapidly proliferating. This constellation of features places these cells at heightened risk for tumor development. Thus, clinical interventions targeting LMP1-expressing MZ B cells should dramatically decrease the incidence of IL in immunosuppressed individuals. Our study is significant in identifying a new reservoir of EBV-infected cells that could give rise to the lymphomas that characteristically develop in immunosuppressed individuals—we have identified these potential tumor precursors as a plausible target for therapeutic intervention. Our findings also provide the first explanation for the biological role of the LCL phenotype and identify the closest normal *in vivo* correlate of these cells. In doing so, we describe and identify a novel site of EBV persistence, independent of the established GC model (GCM) of EBV persistence.

A key caveat is the current lack of technology to perform definitive single cell analysis on *in vivo* EBV-infected human B cells—a technological barrier that constrains our ability to resolve the heterogeneity of viral and host/human transcript and protein expression within individual infected cells. Future development of experimental strategies/systems to selectively isolate and significantly enrich individual *in vivo* EBV-infected cells from chronic EBV carriers would allow for integrated single cell RNA and DNA sequencing, spatial transcriptomics, and proteomic

profiling—combined with immunofluorescence, flow cytometry, fluorescence in situ hybridization (FISH), Western blotting, and blocking assays. We anticipate that such an analysis will reveal constitutive NF-κB signaling and somatic hypermutation signatures in EBV-infected MZ B cells, as observed in ILs/LCLs. Silencing this NF-κB signaling signature specifically in isolated EBV-infected MZ B cells would likely halt their growth as observed in LCLs/ILs. These future studies would re-echo, reinforce, build upon, and extend the translational relevance of our current findings using systems yet to be developed for *in vivo* human EBV research. Additionally, without single cell resolution, it remains to be determined if some EBV-infected MZ B cells could exit the growth program. Our combined EBV gene expression, Ki67, and BCL6 data suggest that a minority of these cells may be able to turn off the growth program and potentially access the GC, and this could have crucial implications on EBV persistence and EBV driven lymphoproliferative disease. This will warrant further studies when single-cell technologies for such *in vivo* human work are developed. We use the term "growth program" when all the growth program genes are expressed based on our analysis, though the precise expression of EBV and host/human transcripts and proteins in individual infected B cells remain unknown. Moreover, the level of EBV gene expression in the cell lines we employed for our real time RT PCR sensitivity assays might not fully reflect the *in vivo* landscape.

Once advancements are made to specifically isolate, enrich and deeply characterize individual *in vivo* EBV-infected cells, we expect to uncover the regulation of EBV and host/human transcripts and proteins in EBV-infected B cells *in vivo* in humans and their clinical consequences—facilitating disease prediction, while enabling rationally designed clinical interventions.

Secondly, regarding our EBV genome copy assay, while it is possible that what occurs in EBV-infected B cells *in vivo* parallels B cells infected *in vitro*, this is an assumption. Nevertheless, this

assay remains the only available method that gives information about the division history of EBV-infected cells *in vivo*. When integrated with the Ki67 data—an independent and more direct indicator of current or active proliferation—our results indicate that EBV-infected MZ B cells have a history of multiple divisions and are actively proliferating, in contrast to EBV-infected naïve B cells, which have undergone few divisions.

Lastly, the current technological barriers in studying EBV biology *in vivo* in humans, and the incomplete understanding of EBV biology, underscore the urgent need for continued and deeper investigation into the cellular origins of various EBV-associated lymphomas. Nevertheless, our identification of GC-independent, EBV-infected tonsillar MZ B cells as the closest normal *in vivo* counterparts of ILs/LCLs provides critical insight into EBV persistence and lymphomagenesis—marking a foundational advance, and a compelling avenue for future mechanistic and translational research, including targeted therapeutic development.

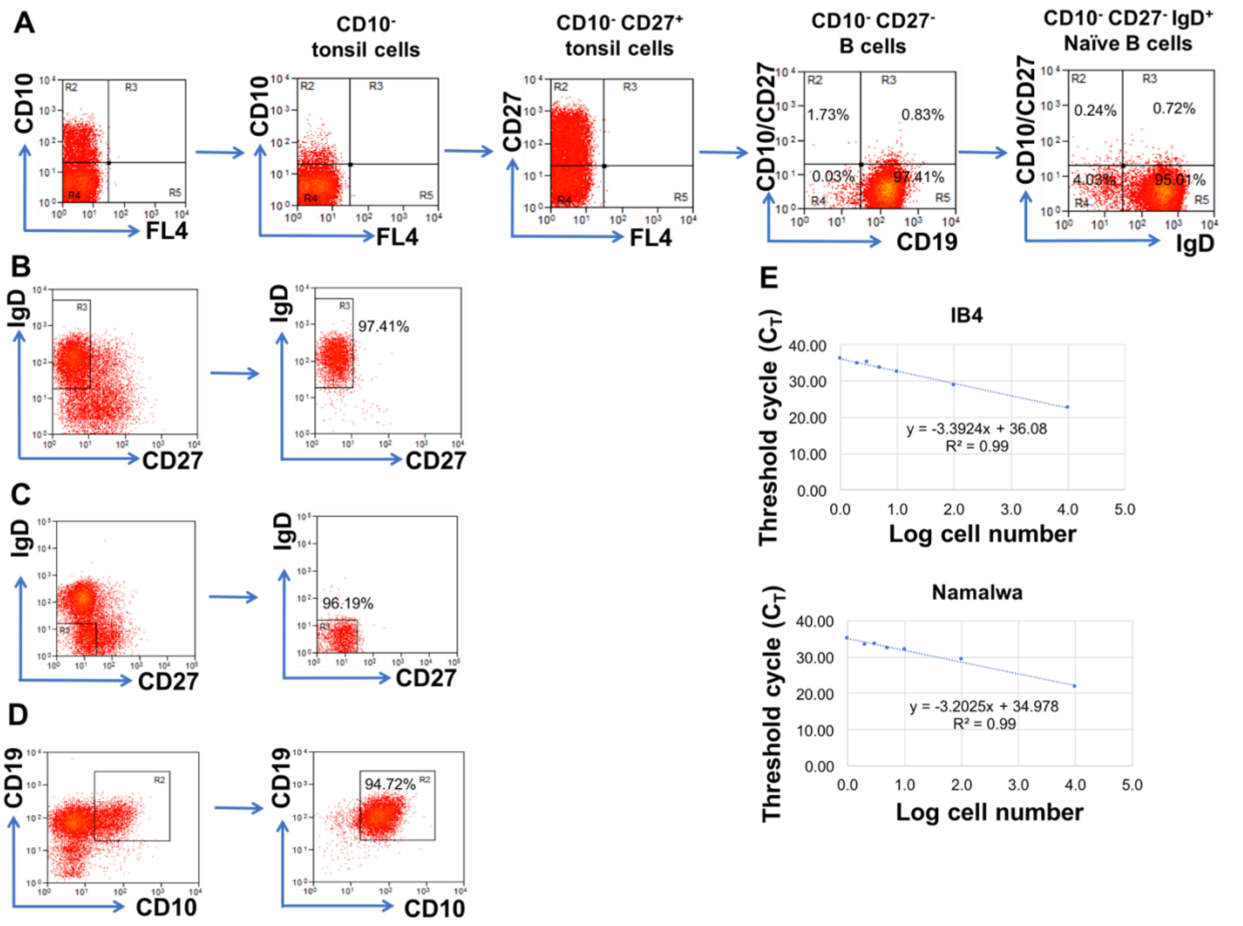

Fig. S1. Alternative isolation of naïve and GC B cells and sensitivity of EBV DNA PCR for the W repeat region of EBV (WPCR). (A and B) Alternate isolation of tonsillar naïve B cells. (A) IgD^+ $CD27^-$ $CD10^-$ naïve B cells were isolated by stepwise column fractionation. CD10 positive cells were first depleted followed by the depletion of CD27 positive cells from negatively selected tonsil B cells. B cells ($CD19^+$) were stained with IgD to check purity. (B) Alternatively, naïve B cells were isolated by FACs analysis and purity assessed by FACs. (C and D) Alternate isolation of tonsillar germinal center B cells. IgD^- $CD27^-$ (C) or $CD19^+$ $CD10^+$ (D) germinal center B cells were isolated by FACs analysis and purity assessed FACs. (E) WPCR standard curve of IB4 lymphoblastoid cell line (top), and Namalwa Burkitt's lymphoma cell line (bottom), with the C_T value plotted against the log value of respective cell numbers. Five replicates of specific cell numbers from each cell line were sorted by FACs analysis; 10000 cells, 100 cells, 10 cells, 5 cells, 3 cells, 2 cells, and 1 cell. Cell lysates were made followed by WPCR analysis and the generation of a standard curve showing the equation of the regression line and the R^2 values. The standard curves indicate that our WPCR assay is sensitive down to one single infected cell/well. N.B. FL represents fluorescent channel.

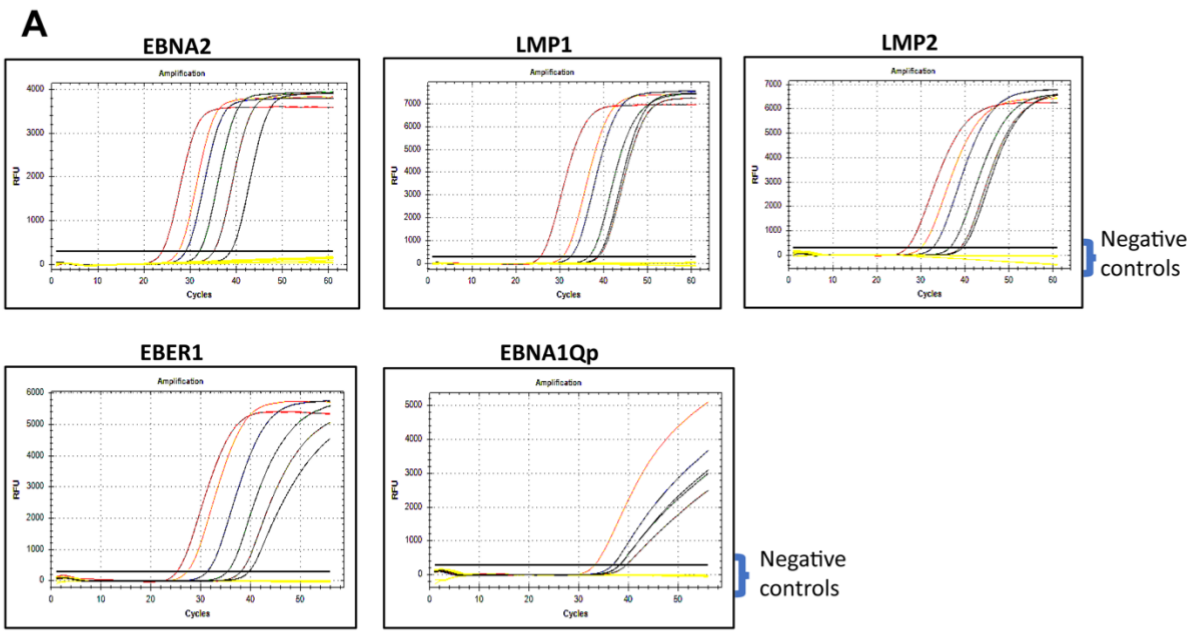

B

Validation of EBV latent gene expression in cell lines by real time RT PCR (Taqman)

	EBV latent gene expression						
Cell line	EBNA2	LMP1	LMP2	EBNA1	EBNA1Qp	EBER1	EBV program
IB4	+	+	+	+	-	+	Growth
SP-LCL-LS	+	+	+	ND	ND	+	Growth
Rael	-[a]	-[b]	-[b]	+	+	+	EBNA1-only

[a] Trace amount of gene transcript observed was negligible.
[b] Trace amount of gene transcript observed was ≤ 1% of LCL value.
+, Signifies positive, i.e. a particular gene transcript is present.
-, Signifies negative, i.e. a given gene transcript is absent.
ND, Signifies not done, i.e. not all genes were tested for all cell lines.

Fig. S2. Sensitivity controls and specificity of the real time RT PCR (Taqman) analysis. (A) EBV positive cell lines were spiked at specific numbers into a standard number of EBV negative tonsil cells or CB60 negative control cell lines before RNA extraction or synthesis of cDNA (Bio-Rad iScript). Independent replicates (2-3) of each cell number were tested in duplicates. IB4 was used as controls for EBNA2, LMP1 and LMP2. Raji was used as control for EBER1 and Akata EBV$^+$ (stimulated with anti-Ig antibody) was used as control for EBNA1Qp. The resultant RNA samples were subjected to real time RT PCR. The amplification plots indicate change in FAM fluorescence. The cell numbers used were 10^5 (red), 10^4 (orange), 10^3 (blue), 10^2 (green), 10 (brown) and 1 (black). Yellow indicates negative controls; 10^4 CB60, the DNAse treated RNA of $10^5/10^4$ IB4, Akata EBV$^+$, and Raji, as well as no template controls. (B) Specific EBV latent gene expression programs can be discriminated with real time RT PCR assays. IB4 LCL and spontaneous LCL SP-LCL-LS treated as in (A) indicate the EBV growth program. Rael treated as

in (A) indicates the EBNA1-only program. The results are shown in linear scale so that the amplification plots for the negative controls are visible.

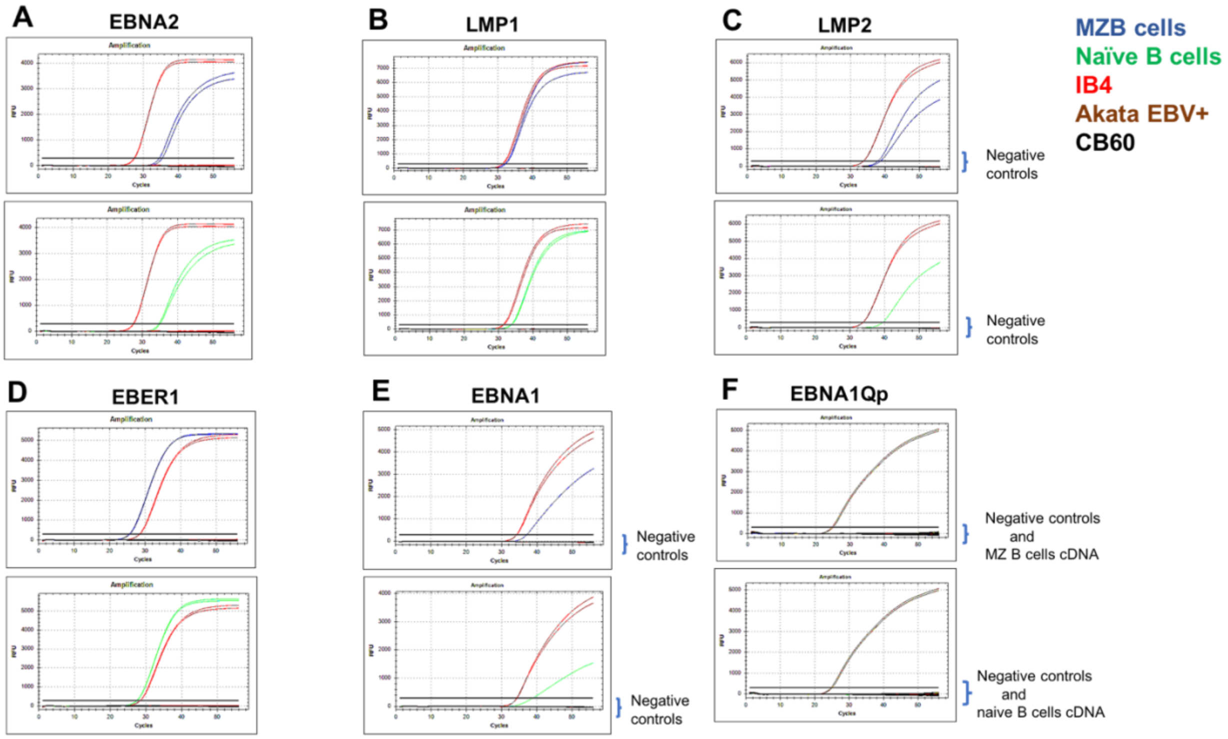

Fig. S3. Detection of EBV transcripts in tonsil B cell subsets by real time RT PCR (Taqman) analysis indicates that tonsillar marginal zone B cells express the growth program. (A – F) Marginal zone B cells (IgD^{+} CD27^{+}, blue), naïve B cells [IgD^{+} CD27^{-} (CD10^{-}), green] that contained specific numbers of EBV-infected cells (~70) were fractionated from tonsil B cells. A specific number of IB4 cell line (200 cells, red), was used as positive control for all latent gene transcripts except EBNA1Qp. Akata EBV^{+} cell line (5x10^{4}, brown) was used as positive control for EBNA1Qp. CB60 cell line (black) was used as EBV negative controls. Where necessary, a standard number of CB60 negative control cells was also used as filler cells. Total RNA was prepared, and cDNA was synthesized with Superscript IV First-Strand system (Invitrogen). Real time RT PCR analysis was performed for the expression of the following EBV latent gene transcripts: (A) EBNA2 (B) LMP1 (C) LMP2 (D) EBER1 (E) EBNA1 and (F) EBNA1Qp. Negative controls: that is, the no reverse transcriptase controls of IB4, Akata EBV^{+} and CB60, or CB60 cDNA, are shown below the threshold cycle line (blue line). The results are shown in linear scale so that the amplification plots for the negative controls are visible.

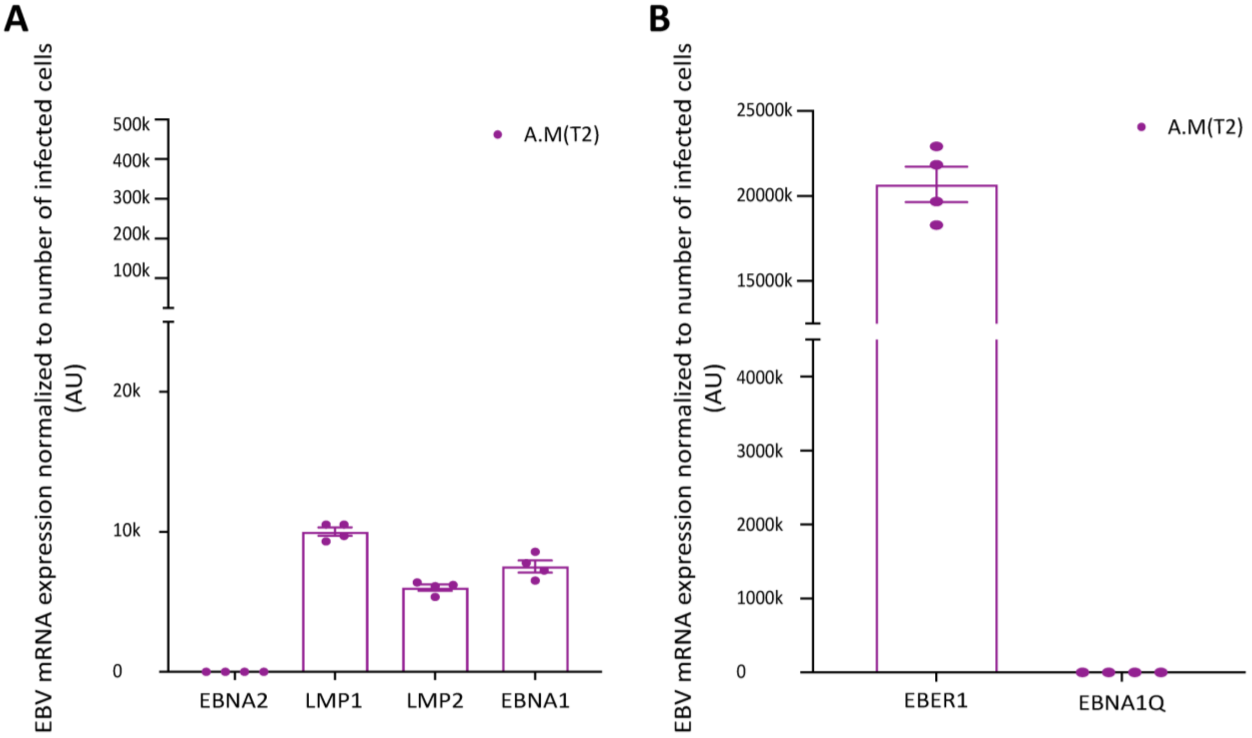

Fig. S4. Real time RT PCR (Taqman) analysis of spontaneously EBV-infected lymphoblastoid cell line A.M(T2) reveals undetectable or low EBNA2 mRNA expression. (A and B) Two hundred (200) cells from the spontaneously EBV-infected lymphoblastoid cell line A.M(T2) were spiked into a standard number of EBV negative tonsil cells or CB60 negative control cell lines before RNA extraction and synthesis of cDNA with Superscript IV First-Strand system (Invitrogen). EBV gene expression was analyzed by real time RT PCR (Taqman) on each independent sample replicate in duplicates (n = 4).

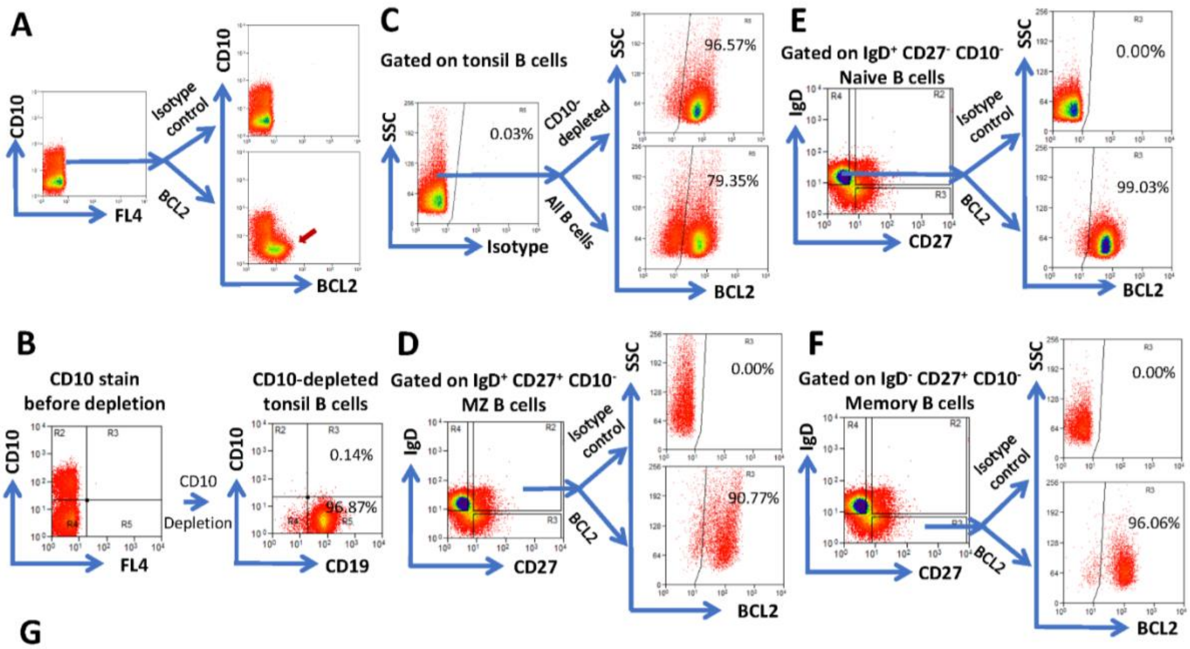

G

Frequency of EBV-infected cells in CD10-depleted ($CD10^-$) tonsillar BCL2 positive marginal zone B cells			
	Number of virus-infected cells/10^7 B cells [a]		
Tonsil	IgD^+ $CD27^+$ $CD10^-$	IgD^+ $CD27^+$ $CD10^-$ $BCL2^+$	IgD^+ $CD27^+$ $CD10^-$ $BCL2^-$
7	3560	4796	ND
12	402	275	ND

[a] The number/frequency of EBV-infected cells was determined by limiting dilution DNA PCR as in Fig 2.
ND, This signifies not done, i.e. $BCL2^-$ marginal zone B cells were not tested.
N.B., Each tonsil represents a different donor.

Fig. S5. Tonsillar marginal zone (IgD^+ $CD27^+$ $CD10^-$) B cells are BCL2 positive. (A) Flow cytometric analysis for the expression of the pro-survival regulatory protein BCL2 in tonsil non-GC ($CD10^-$) B cells (positive control). Purified tonsil B cells were fixed, permeabilized and stained for the intracellular BCL2 (bottom) or isotype control (top). These cells were then washed and stained for the extracellular GC-specific marker CD10. The red arrow indicates $BCL2^+$ non-GC B cells. (B) Depletion of $CD10^+$ cells from tonsil B cells by column fractionation. (C) CD10-depleted tonsil B cells (top) as in (B) or whole tonsil B cells (bottom) were stained for the intracellular marker BCL2 or isotype control. This confirms results in (A) showing that BCL2 is expressed in non-GC B cells. (D) CD10-depleted tonsil B cells as in (B) were stained for the intracellular marker BCL2 (bottom) or isotype control (top) and the extra cellular markers IgD and CD27 as described in (A). $BCL2^+$ cells were then sorted from the IgD^+ $CD27^+$ $CD10^-$ marginal zone B-cell population. Total IgD^+ $CD27^+$ $CD10^-$ marginal zone B cells were also sorted. (E) CD10-depleted tonsil B cells stained as in (D) were gated on BCL2/isotype control expressing IgD^+ $CD27^-$ $CD10^-$ naïve B cells. (F) CD10-depleted tonsil B cells stained as in (D) were gated on BCL2/isotype control expressing IgD^- $CD27^+$ $CD10^-$ memory B cells. (G) The frequency of EBV-infected cells in total marginal zone B cells and $BCL2^+$ marginal zone B cells was assessed based on limiting dilution DNA PCR. The Poisson statistics was applied and then the absolute frequency of EBV-infected cells was

calculated. Results of two different tonsils are shown (n = 2). N.B., FL represents fluorescent channel.

Table S1. The phenotype of lymphoblastoid cell lines (LCLs) derived from *in vitro* infection of naïve B cells correlates with marginal zone (MZ), but no other B cell subsets

Marker	LCL	MZ	Naive	GC	Memory
IgD	+	+	+	-	-
CD27 (Memory)	+	+	-	-/+	+
CD10 (GC)	-	-	-	+	-
BCL6 (GC)	-	-	-	+	-
AID	+	?	-	+	-
SHM	+	+	-	+	+
EBV latency program when infected *in vivo*	Growth	?	Growth?	Default	EBNA1-only[a]/0

[a] EBNA1 expression is from the Cp promoter in the growth program but from the Qp promoter (a different promoter) in the default and the EBNA1-only program.

+, This signifies positive, that is, a particular marker is present.

-, This signifies negative, that is, a given marker is absent.

?, This signifies uncertain or unknown.

Adapted from (1).

Table S2. Frequency of EBV-infected cells in tonsillar naïve B cells/10^7 B cells [a]

	Number of experiments [b]				
Tonsil	1	2	3	4	Mean
1	200	400	240	230	268
14	100	70	50	ND	73
16	100	100	120	ND	107

[a] The number/frequency of EBV-infected cells was determined by limiting dilution DNA PCR as in Fig. 2.

[b] These values represent separate determinations made on the same tonsil at independent/different times.

ND, Signifies not done, that is, not all donor naïve B cell compartments were tested four independent times.

N.B., Each tonsil represents a different donor.

Table S3. Frequency of EBV-infected cells in tonsillar memory B cells/10^7 B cells [a]

	Number of experiments [b]					
Tonsil	1	2	3	4	5	Mean
1	3,120	2,010	1,005	2,840	2,300	2,255
14	2,010	1,005	ND	ND	1,230	1,415
15	1,230	1,290	ND	ND	1,500	1,340

[a] The number/frequency of EBV-infected cells was determined by limiting dilution DNA PCR as in Fig. 2.

[b] These values represent separate determinations made on the same tonsil at independent/different times.

ND, Signifies not done, that is, not all donor memory B cell compartments were tested five independent times.

N.B., Each tonsil represents a different donor.

Table S4. Frequency of EBV-infected cells in tonsillar germinal center B cells/10^7 B cells [a]

	Number of experiments [b]				
Tonsil	1	2	3	4	Mean
1	1,005	1,410	1,005	ND	1,140
14	100	200	100	ND	133
15	200	160	170	155	171
17	10	10	7	ND	9

[a] The number/frequency of EBV-infected cells was determined by limiting dilution DNA PCR as in Fig. 2.

[b] These values represent separate determinations made on the same tonsil at independent/different times.

ND, Signifies not done, that is, not all donor germinal center B cell compartments were tested four independent times.

N.B. 1., Each tonsil represents a different donor.

N.B. 2., The germinal center B cell data from tonsil 1, 14 and 15 have been published previously (2)

Table S5. Frequency of EBV-infected cells in tonsillar marginal zone B cells/10^7 B cells [a]

	Number of experiments [b]			
Tonsil	1	2	3	Mean
Tonsil 12	402	487	360	416
Tonsil 18	376	317	ND	347
Tonsil 21	173	107	ND	140
Tonsil 22	82	59	53	65

[a] The number/frequency of EBV-infected cells was determined by limiting dilution DNA PCR as in Fig. 2.

[b] These values represent separate determinations made on the same tonsil at independent/different times.

ND, Signifies not done, that is, not all donor marginal zone B cell compartments were tested three independent times.

N.B., Each tonsil represents a different donor.

Table S6. Number of B cells for frequency of EBV-infected cells in CD10-depleted ($CD10^-$) tonsillar Ki67 positive marginal zone B cells assay [a]

	Number of B cells assayed for the level of EBV-infected B cells in B cell subpopulations [b]		
Tonsil	IgD^+ $CD27^+$ $CD10^-$	IgD^+ $CD27^+$ $CD10^-$ $Ki67^+$	IgD^+ $CD27^+$ $CD10^-$ $Ki67^-$
6	ND	3.75×10^4	1.81×10^5
7	ND	3.41×10^3	4.03×10^4
7 [c]	ND	2.89×10^4	2.31×10^5
8	ND	1.94×10^4	1.49×10^5
12	7.94×10^5	1.25×10^5	1.13×10^6
18	1.89×10^5	5.18×10^4	8.00×10^5

[a] B cells assayed for the level of EBV-infected B cells were sorted by flow cytometry as described in the methods.

[b] Approximate number of B cells assayed for the level of EBV-infected B cells, with the aid of limiting dilution DNA PCR as in Fig. 2, in respective B cell subpopulations.

[c] Tonsils from the same patient was assayed again independently on separate occasions.

ND, Signifies not done, that is, not all marginal zone B cells were tested for all tonsils.

N.B., Each tonsil represents a different donor.

Table S7. Number of B cells for frequency of EBV-infected cells in CD10-depleted ($CD10^-$) tonsillar AID positive marginal zone B cells assay [a]

	Number of B cells assayed for the level of EBV-infected B cells in B cell subpopulations [b]		
Tonsil	IgD^+ $CD27^+$ $CD10^-$	IgD^+ $CD27^+$ $CD10^-$ AID^+	IgD^+ $CD27^+$ $CD10^-$ AID^-
5	ND	1.10×10^4	6.09×10^4
6	8.40×10^5	4.27×10^4	4.20×10^5
7	ND	1.57×10^4	1.40×10^5
18	ND	2.93×10^4	2.52×10^5

[a] B cells assayed for the level of EBV-infected B cells were sorted by flow cytometry as described in the methods.

[b] Approximate number of B cells assayed for the level of EBV-infected B cells, with the aid of limiting dilution DNA PCR as in Fig. 2, in respective B cell subpopulations.

ND, Signifies not done, that is, not all marginal zone B cells were tested for all tonsils.

N.B., Each tonsil represents a different donor.

Table S8. Number of B cells for frequency of EBV-infected cells in CD10-depleted ($CD10^-$) tonsillar BCL6 negative marginal zone B cells assay [a]

	Number of B cells assayed for the level of EBV-infected B cells in B cell subpopulations [b]		
Tonsil	IgD^+ $CD27^+$ $CD10^-$	IgD^+ $CD27^+$ $CD10^-$ $BCL6^+$	IgD^+ $CD27^+$ $CD10^-$ $BCL6^-$
4	9.17×10^4	4.17×10^3	1.68×10^5
7	ND	4.72×10^3	3.00×10^5
12	2.24×10^5	9.73×10^3	5.37×10^5
13	ND	3.96×10^4	1.17×10^6
19	1.00×10^5	4.57×10^3	1.24×10^5
20	ND	1.01×10^4	6.78×10^5
22	ND	1.40×10^4	1.07×10^6

[a] B cells assayed for the level of EBV-infected B cells were sorted by flow cytometry as described in the methods.

[b] Approximate number of B cells assayed for the level of EBV-infected B cells, with the aid of limiting dilution DNA PCR as in Fig. 2, in respective B cell subpopulations.

ND, Signifies not done, that is, not all marginal zone B cells were tested for all tonsils.

N.B., Each tonsil represents a different donor.

Table S9. Details of primers and probe combination for EBV assays

Target	Forward (For), Reverse (Rev) Primers, and FAM-TAMRA Taqman probe (Probe)
EBER1	For: ACCGAAGACGGCAGAAAGC<br>Rev: CCTACGCTG CCCTAGAGGTTT<br>Probe: ACAGACACCGTCCTCACCACCCG
LMP1	For: ACCACGACACACTGATGAACAC<br>Rev: CTAGAATCGTCGGTAGCTTGTTGA<br>Probe: ACTCCCTCCCGCACCC
LMP2	For: AGCTGTAACTGTGGTTTCCATGAC<br>Rev: GCCCCC TGGCGAAGAG<br>Probe: CTGCTGCTACTGGCTTTCGTCCTCTGG
EBNA1	For: TGAGTCGTCTCCCCTTTGGA<br>Rev: CCTTAGTGGGCCAGGTTGTG<br>Probe: ATGGCCCCTGGACCC
EBNA2	For: CACCACTACTCACGGTACTACAAAGG<br>Rev: CATTGATTGGTCTGGCACATG<br>Probe: CCACCACGCATGCATCTCCCTGT
EBNA1Qp (3)	For (Q): GTGCGCTACCGGATGGC<br>Rev (UK): CATGATTCACACTTAAAGGAGACGG<br>Probe (U): TCCTCTGGAGCCTGACCTGTGATCG
W	For: AGTGGGCTTGTTTGTGACTTCA<br>Rev: GGACTCCTGGCGCTCTGAT<br>Probe: TTACGTAAGCCAGACAGCAGCCAATTGTC

SI References

1. Thorley-Lawson DA. 2015. EBV Persistence--Introducing the Virus. Curr Top Microbiol Immunol 390:151-209.
2. Torgbor C, Awuah P, Deitsch K, Kalantari P, Duca KA, Thorley-Lawson DA. 2014. A multifactorial role for P. falciparum malaria in endemic Burkitt's lymphoma pathogenesis. PLoS Pathog 10:e1004170.
3. Bell AI, Groves K, Kelly GL, Croom-Carter D, Hui E, Chan AT, Rickinson AB. 2006. Analysis of Epstein-Barr virus latent gene expression in endemic Burkitt's lymphoma and nasopharyngeal carcinoma tumour cells by using quantitative real-time PCR assays. J Gen Virol 87:2885-90.
